# Direct Anthelmintic Effects of Condensed Tannins from Diverse Plant Sources against *Ascaris suum*

**DOI:** 10.1371/journal.pone.0097053

**Published:** 2014-05-08

**Authors:** Andrew R. Williams, Christos Fryganas, Aina Ramsay, Irene Mueller-Harvey, Stig M. Thamsborg

**Affiliations:** 1 Department of Veterinary Disease Biology, Faculty of Health and Medical Sciences, University of Copenhagen, Frederiksberg, Denmark; 2 Chemistry and Biochemistry Laboratory, School of Agriculture, Policy and Development, University of Reading, Reading, United Kingdom; Swiss Tropical and Public Health Institute, Switzerland

## Abstract

*Ascaris suum* is one of the most prevalent nematode parasites in pigs and causes significant economic losses, and also serves as a good model for *A. lumbricoides*, the large roundworm of humans that is ubiquitous in developing countries and causes malnutrition, stunted growth and compromises immunity to other pathogens. New treatment options for *Ascaris* infections are urgently needed, to reduce reliance on the limited number of synthetic anthelmintic drugs. In areas where *Ascaris* infections are common, ethno-pharmacological practices such as treatment with natural plant extracts are still widely employed. However, scientific validation of these practices and identification of the active compounds are lacking, although observed effects are often ascribed to plant secondary metabolites such as tannins. Here, we extracted, purified and characterised a wide range of condensed tannins from diverse plant sources and investigated anthelmintic effects against *A. suum in vitro.* We show that condensed tannins can have potent, direct anthelmintic effects against *A. suum*, as evidenced by reduced migratory ability of newly hatched third-stage larvae and reduced motility and survival of fourth-stage larvae recovered from pigs. Transmission electron microscopy showed that CT caused significant damage to the cuticle and digestive tissues of the larvae. Furthermore, we provide evidence that the strength of the anthelmintic effect is related to the polymer size of the tannin molecule. Moreover, the identity of the monomeric structural units of tannin polymers may also have an influence as gallocatechin and epigallocatechin monomers exerted significant anthelmintic activity whereas catechin and epicatechin monomers did not. Therefore, our results clearly document direct anthelmintic effects of condensed tannins against *Ascaris* and encourage further *in vivo* investigation to determine optimal strategies for the use of these plant compounds for the prevention and/or treatment of ascariosis.

## Introduction

The closely related worms *Ascaris suum* and *A. lumbricoides* are among the most prevalent nematode parasites in pigs and humans, respectively. More than 500 million people are estimated to be infected with *A. lumbricoides,* resulting in significant morbidity, stunted development and malnutrition, mainly in children [1]. *Ascaris suum* has a high prevalence in pigs in both developed and developing countries, resulting in significant economic penalties for pig farmers [2], [3]. Moreover, *A. suum* is considered a zoonotic parasite and hybridization between *A. suum* and *A. lumbricoides* has been reported [4]. In addition, *Ascaris* infection also predisposes hosts to co-infection with bacteria and/or protozoa and compromises vaccine efficacy against other pathogens [5], [6].

At present, *Ascaris* control is based mainly on mass treatment with synthetic anthelmintic drugs. In the long-term this is not sustainable – re-infection after annual or bi-annual drug treatment is more or less unavoidable due to the long-lived and resistant eggs which survive for many years in the environment [7], and the reliance on a limited number of related compounds (mainly albendazole and mebendazole) for mass drug administration against *A. lumbricoides* means that the threat of drug resistance is an on-going concern [1]. Moreover, the use of synthetic drugs is often not feasible for *A. suum* control in many pig production systems. Small holder farmers in many developing countries often do not have access to expensive anthelmintic drugs and, in developed countries, many organic and low-input farms are not able to prophylactically treat animals with synthetic drugs. Therefore, there is an urgent need to investigate alternative and/or complementary options for the control of these parasites.

The use of natural plant extracts as anthelmintics has been practiced in many indigenous cultures for centuries. Indeed, in many developing countries ethno-medicine is still the primary treatment option for many parasitic diseases [8], [9]. The use of plant extracts has several obvious advantages that make them attractive for use in developing countries – low cost, access to large amounts of raw material and easy integration into traditional cultural practice [8]–[10]. However, scientific validation of these traditional treatments is lacking. Most studies that have investigated the anthelmintic potential of traditional medicinal plants have focused on crudely prepared extracts from a limited selection of plants, with only a summary analysis of the chemical constituents, and no further investigation of the active compounds [11]. Cleary, there is a need for more systematic studies to identify and validate the use of plants as anthelmintics.

**Figure 1:**
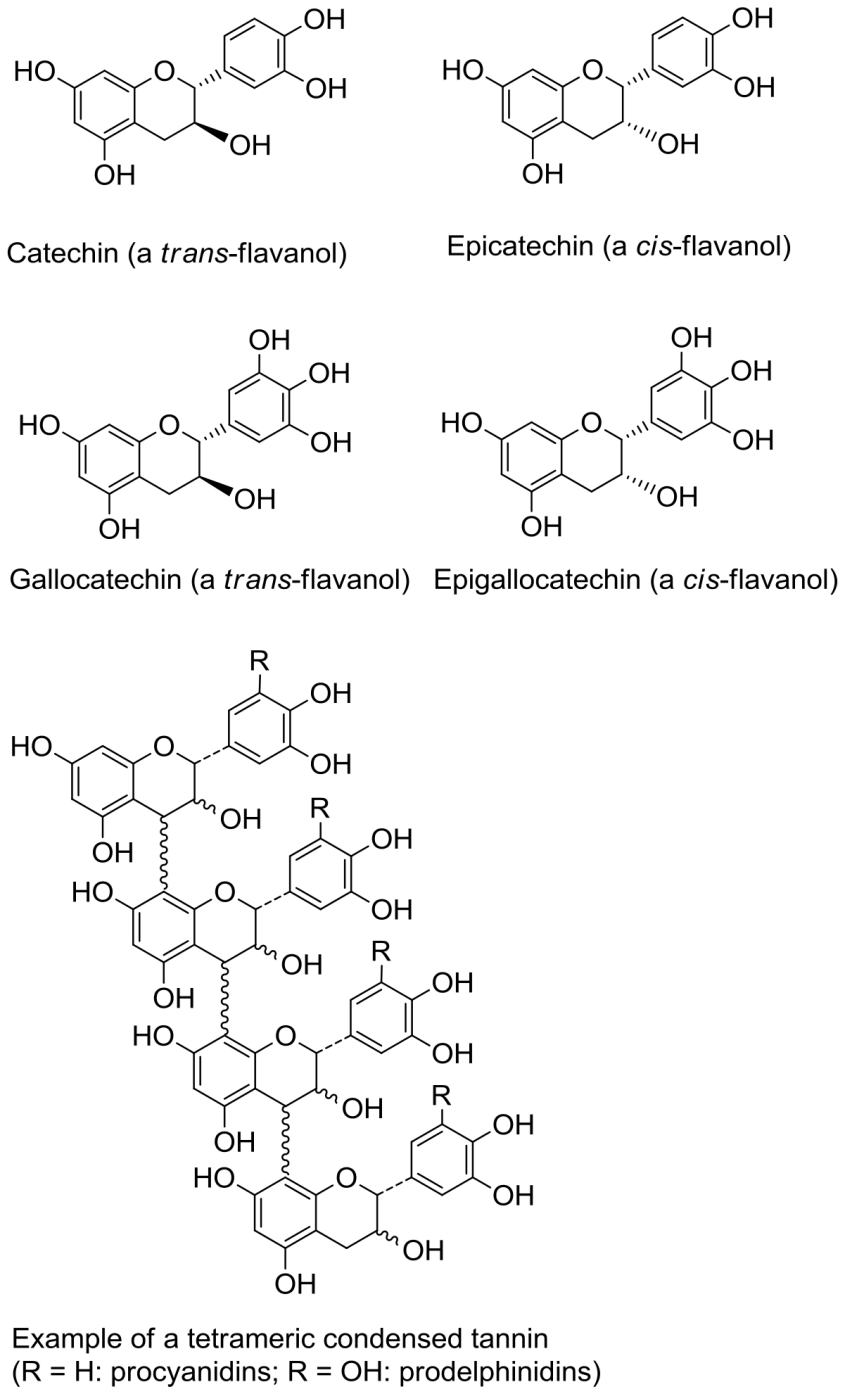
Structures of flavanol monomeric subunits and an example of a tetrameric condensed tannin.

Condensed tannins (CT) are a diverse group of plant secondary metabolites commonly found in leaves, roots, nuts and fruits from a wide-range of different plant sources in both tropical and temperate areas [12]. Condensed tannins are formed by the polymerisation of monomeric flavan-3-ols. The structure of CT can vary considerably according to the degree of polymerisation, and the nature of the monomeric flavan-3-ol units; these being either catechin (C) and its *cis* isomer epicatechin (EC) which make up procyanidins, or gallocatechin (GC) and epigallocatechin (EGC), which make up prodelphinidins (Figure 1). Condensed tannins are well-known for their antimicrobial properties [13], with reports of potent effects against, amongst others, *Candida albicans* [14], *Trypanosoma brucei* [15], and *Leishmania donovani* [16]. In addition, a number of studies conducted with nematode parasites of ruminant livestock have demonstrated direct anthelmintic effects of extracts from tannin-containing plants in *in vitro* assays, with *in vivo* verification of these results being reported in some studies [17]. Furthermore, CT have been shown to have *in vitro* and *in vivo* efficacy in rodent models of hookworm and pinworm [18], [19], and are speculated to be responsible for the anthelmintic effects of some traditional medicinal plants against *Ascaris* [20], [21] although definitive evidence of this has not yet been provided. Therefore, studies to determine whether CT have direct anthelmintic effects against *Ascaris* are clearly warranted. Moreover, it is apparent that the anthelmintic effects of CT that thus far have been reported in other livestock parasites such as *Haemonchus contortus* are influenced by tannin structure [22], and given the diverse nature of CT from different plant sources [23], the relationship between anthelmintic activity and tannin structure also requires investigation.

Here, we extracted and characterised CT with a wide range of structural diversity from a variety of different plant sources, and carried out a detailed analysis of direct anthelmintic effects against *A. suum in vitro*. We show that, similarly to some other nematode species, CT can have potent anthelmintic effects against *A. suum*, and that the strength of the anthelmintic activity is likely related to a complex set of factors including degree of polymerization and the identity of the monomeric flavanol units in the CT molecules. Therefore, our results provide the first clear evidence of the direct anthelmintic effects of CT against *A. suum* and encourage further *in vivo* evaluation of CT-containing plant extracts for the treatment of ascariosis.

## Methods

### Ethics Statement

All animal experimentation was approved and carried out according to the guidelines of the Danish Animal Experimentation Inspectorate (Licence number 2010/561-1914). Protocols were approved by the Animal Ethics Committee, Department of Experimental Medicine, University of Copenhagen.

### Materials

Flavanol monomers (catechin, epicatechin, gallocatechin and epigallocatechin) were purchased from Sigma-Aldrich (Denmark). Plant samples were chosen in order to provide a wide variety of CT structural characteristics and were obtained as follows: cocoa beans were purchased from ‘Detox your World’ company (Great Yarmouth, UK), hazelnut skins were provided by Dr H. Hoste (INRA Toulouse, France), pine bark (*Pinus sylvestris*) was provided by Dr M. Karonen (University of Turku, Finland), whole sainfoin (*Onobrychis viciifolia,* var. Esparsette) plants were provided by Mr P. Davy (Barham, Kent, UK), leaves from blackcurrant (*Ribes nigrum*) and redcurrant (*Ribes rubrum*) bushes were collected from Hildred’s Pick-Your-Own Farm (Goring-upon-Thames, UK), and white clover (*Trifolium repens*) flowers from NIAB (Cambridge, UK).

### Extraction and Fractionation of Tannins from Plants

The freeze-dried, ground cocoa beans (125 g) were defatted three times with hexane (600 mL) at room temperature for 30 min; the extract was filtered and hexane discarded. The defatted powder was air dried for 3 to 4 hours and kept at room temperature. All plant samples (50 g) were treated with acetone/water (7∶3; v/v) to extract tannins [24]. Solutions were concentrated and aqueous extracts were freeze-dried. Portions of the freeze-dried extracts were dissolved in water and loaded onto a Sephadex-LH20 column. The column was rinsed with water, low molecular weight tannins were eluted with acetone/water (3∶7; v/v; Fraction 1) and higher molecular weight tannins with acetone/water (1∶1; v/v; Fraction 2).

### Tannin Analysis by HPLC

Tannins in extracts and fractions (except for cocoa beans – see below) were characterized by thiolytic degradation according to Gea *et al.* [24] with slight modification of the HPLC analysis. Samples (20 µL) were injected into the Gilson HPLC system connected to an ACE C18 column (3 µm; 250×4.6 mm; Hichrom Ltd; Theale; UK) fitted with a corresponding ACE guard column kept at room temperature. The flow rate was 0.75 mL min^−1^ using 1% acetic acid in water (solvent A) and HPLC-grade acetonitrile (solvent B). The following gradient programme was employed: 0–35 min, 36% B; 35–40 min, 36–50% B; 40–45 min, 50–100% B; 45–50 min, 100–0% B; 50–55 min, 0% B. HPLC-analysis provides information on percentage of flavanols (catechin, epicatechin, gallocatechin and epigallocatechin) in terminal and extension units, tannin content, mean degree of polymerization (mDP) or average polymer size, percentage of prodelphinidin and procyanidin tannins and percentage of *cis*- and *trans*-flavanol units in tannins [24] (see also Figure S1).

Cocoa bean tannins were characterised by liquid chromatography-mass spectrometry (LC-MS). Terminal and extension units after thiolysis were identified on a HPLC Agilent 1100 series system consisting of a G1379A degasser, G1312A binary pump, a G1313A ALS autoinjector, a G1314A VWD UV detector and a G1316A column oven and API-ES instrument Hewlett Packard 1100 MSD Series (Agilent Technologies, Waldbronn, Germany) using an ACE C18 column (3 µm; 250×4.6 mm; Hichrom Ltd; Theale; UK) fitted with ACE guard column at room temperature. Data were acquired with ChemStation software (version A 10.01 Rev. B.01.03). The injection volume was set to 20 µL and flow rate to 0.75 mL/min. The sample was eluted using a gradient of 1% acetic acid in MilliQ H_2_O (solvent A) and HPLC-grade acetonitrile (solvent B) as follows: 0–35 min, 36% B; 35–40 min, 36–50% B; 40–45 min, 50–100% B, which was followed by 45–55 min, 100–0% B; 55–60 min, 0% B. UV-vis spectra were recorded at 280 nm. MS spectra recorded in the negative ionisation scan mode between *m/z* 100 and 1000 used the following conditions: 3000 V for capillary voltage, nebuliser gas pressure at 35 psig, drying gas at 12 mL/min and dry heater temperature at 350°C. MS spectra recorded in the positive ionization scan were with the same parameters as for negative but the capillary voltage was 3000 V. Terminal and extension units were identified by their retention times and molecular masses.

### Parasites

To obtain viable third-stage larvae (L3), gravid *A. suum* female worms were collected from the intestine of pigs at a local slaughterhouse (Danish Crown, Ringsted, Denmark). Eggs were isolated from the uteri of worms and embryonated for at least two months at room temperature as previously described [25]. The eggs were decoated (removing the outer proteinaceous shell) using NaOH and then stored at 4°C in 1 M H_2_SO_4_. For hatching, eggs were washed thoroughly with 0.9% NaCl to remove the sulphuric acid, and *in vitro* hatching was then achieved by using a method modified from that of Han *et al.* [26]. 1.25 mL of egg solution (5 eggs/µL) was added to 5 mL of 0.9% NaCl and 300 µL of pig bile in a tissue culture flask. The flasks were then incubated overnight on a plate shaker (120 rpm) in an environment of 37°C and 100% CO_2._ The next day, hatched L3 were separated from un-hatched eggs and debris by migration through a 20 µm sieve. The larvae were then suspended in RPMI 1640 media (Gibco) containing HEPES, L-glutamine and 100 µg/U/mL of penicillin/streptomycin and used in the L3 migration inhibition assay (see below).

Fourth-stage larvae (L4) were obtained by infecting two eight-week old pigs each with 10,000 embryonated *A. suum* eggs using a stomach tube. Animals were monitored daily – no clinical signs of ascariosis or any other signs of ill-health were observed. 12 after infection, the pigs were killed by captive bolt pistol and exsanguination, and the entire small intestine was removed, opened and the contents collected. The intestine was thoroughly washed with water, and the contents and washings were then combined and added to an equal amount of 2% agar at 50°C as described previously [27]. Larvae were recovered by migration from the agar into warm saline for 2 hours at 37°C. Larvae were then further cleaned by active migration through three layers of gauze into warm saline. The larvae were transferred to 50 mL centrifuge tubes and thoroughly washed by repeated sedimentation, first in warm saline and then in warm, sterile culture media (RPMI 1640 containing HEPES, L-glutamine, 100 µg/U/mL of penicillin/streptomycin and 0.2 µg/mL amphorectin B). After 90 minutes incubation at 37°C, the larvae were washed a further five times in culture media and then used in the L4 motility inhibition assay (see below).

### Migration Inhibition Assay with Newly Hatched Larvae

The migration inhibition assay was based on the method described by Han *et al.* [26] with some modifications. Extracts and tannin fractions were dissolved in culture media at appropriate concentrations and then 500 µL added to triplicate wells on a 48-well tissue culture plate. Negative and positive controls consisted of media only and 50 µg/mL ivermectin, respectively. 100 larvae were then added to each well and the plate incubated for 16 hours at 37°C in an atmosphere of 5% CO_2_. The motility of larvae was then scored by a single observer on a 0–5 scale outlined by Stepek *et al.* [28], where 5 is fully motile (i.e. identical to the motility at the start of the incubation period) and 0 is completely motionless. Then, 500 µL of 1.6% agar solution at 45°C was added to each well, immediately mixed, and then 500 µL of this mixture was transferred to the corresponding well on another 48-well plate (i.e. two wells for each original triplicate well, each of final volume of 500 µL, 0.8% agar). The agar was allowed to set, and then culture medium was added on top of the gel and the plates placed back in the incubator overnight. Larvae that migrated to the media on the surface of the gel were counted by light microscopy. Numbers from both wells were summed. Larvae began to migrate out of the gel almost immediately and migration was generally complete after around 4 hours – no further migration was observed during the following 24 hours, and the numbers of larvae in the media did not decrease, indicating that larvae remained in the media on the surface of the gel and did not migrate back into the agar. Therefore, the numbers of larvae migrating from the gel was routinely assessed after 16–18 hours (overnight).

Percentage inhibition of migration was calculated relative to the negative control using the formula:
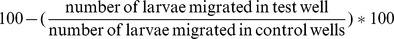



To quantify whether the presence of CT in the media influenced the agar migration assay, in some experiments larvae were washed with RPMI 1640 after the incubation period to remove tannins from the media before the addition of agar – this was done by centrifugation three times for 3 minutes each at 500 g.

### Polyvinylpolypyrrolidone (PVPP) Treatment

PVPP was used to selectively remove tannins from the dissolved extracts. Extracts were dissolved at a concentration of 1 mg/mL before the addition of 50 mg/mL PVPP (Sigma-Aldrich) in a plastic tube. Tubes were incubated overnight at 4°C and precipitated tannins removed by centrifugation at 3000 g for ten minutes. The supernatant was used in the assays. Controls consisted of 1) dissolved extract without PVPP and 2) media alone with 50 mg/mL PVPP. These were incubated overnight in an identical fashion and assayed alongside the PVPP-treated extracts.

### L4 Motility Inhibition Assay

To assess the anthelmintic effects of the tannins against L4 stage parasites, we used a motility inhibition assay similar to that recently described [29], [30]. Five worms were placed into triplicate wells of a 48 well plate with culture media containing either a range of tannin concentrations, 100 µg/mL of either ivermectin or levamisole (positive control), or culture media only (negative control). The plates were then incubated at 37°C in an atmosphere of 5% CO_2_ in air. At 12-hour intervals the motility of the worms was scored using the above-mentioned 0–5 scale. Larvae that scored 0 on two consecutive time-points were considered dead. Dead larvae were removed and fixed for electron microscopy (see below). Control larvae were fixed at the same time for comparison.

### Transmission Electron Microscopy

Worms were washed thoroughly with PBS before fixation in 2% glutaraldehyde in 0.05 M phosphate buffer. The samples were then postfixed, firstly in 1% OsO_4_ and 0.05 M K_3_FeCN_6_ in 0.12 M cacodylate buffer for 2 hours at RT, followed by 1% uranyl acetate for 1 hour at RT. The samples were then dehydrated in an ethanol gradient, infiltrated with epon/propyleneoxide and embedded in 100% expoxy resin and polymerized at 60°C overnight. Samples were then sectioned at 70 nm and stained with uranyl acetate and lead citrate before viewing under a Phillips CM100 transmission electron microscope. Images were obtained with a Olympus Veleta Camera and processed using ITEM software.

### Data Analysis and Statistics

The concentration of tannins in the F1 and F2 fractions needed to induce 50% migration inhibition activity (MIA EC_50_) was estimated by non-linear least squares regression. For each fraction, a variable-slope dose response curve was fitted, with the top of the curve constrained to 100% MIA and the bottom to 0% MIA. Differences between the EC_50_ of the F1 and F2 fractions from the same sample were assessed by extra sum-of-squares F-test, assuming a null hypothesis of equal EC_50_. The effect of PVPP treatment and individual monomers was assessed on arcsine-transformed MIA data using two-way ANOVA with Bonferroni post-hoc testing. Multiple regression was used to investigate associations between MIA EC_50_ values of the F2 tannin fractions and structural characteristics of the tannin molecules (mDP, % monomeric units and % *cis* isomerism). Graphpad Prism 6 was used for all analyses except for the multiple regression, for which SPSS 20.0 was used. *P* values of <0.05 were considered significant.

## Results

### Extracts from Plants Containing Condensed Tannins have Anthelmintic Activity against Ascaris Suum

As a series of contrasting condensed tannins (CT) that differ in polymer size and procyanidin/prodelphinidin proportions cannot be obtained commercially, we extracted tannins from seven common plant sources known to contain different CT types. The extracted tannins were subjected to thiolytic degradation, analysed by HPLC and shown to contain varying amounts of CT of differing flavanol composition (Table S1). From these data, the mean degrees of polymerisation (mDP), PC/PD and *cis*/*trans* ratios were calculated and are summarised in Table 1. To ascertain whether these extracts had activity against *A. suum*, we hatched fully embryonated eggs *in vitro* to obtain viable, infective L3. Larvae were then incubated either in culture media alone or with one of the seven extracts at each of four concentrations ranging from 1 mg/mL to 125 µg/mL. After 16 hours, the larvae incubated in media alone were still fully motile (Figure S2). Incubation with the extracts at concentrations of ≥500 µg/mL reduced motility compared to the negative control, with the exception of sainfoin, where no obvious differences in motility were observed (Figure S2). Notably, at high concentrations (≥500 µg/mL) of all extracts except sainfoin, some dead larvae were observed (up to 15–20% in some extracts at 1 mg/mL). In contrast, dead larvae were never observed in the control group. Thus, it appears that these tannin-containing extracts have anthelmintic activity against *A. suum.*

**Table 1:** Chemical analysis of acetone/water extracts.

Sample	CT (g/100 g extract)	PC : PD ratio	mDP	*cis* : *trans* ratio
**Sainfoin**	12.8	25.3∶74.7	5.5	80.2∶19.8
**Cocoa beans**	13.0	100.0∶0.0	2.9	96.0∶4.0
**Pine bark**	50.8	64.2∶35.8	2.5	79.6∶ 20.4
**Hazelnut skin**	73.8	79.5∶20.5	9.6	49.8∶50.2
**Blackcurrant leaves**	29.3	5.8∶ 94.2	5.4	9.1∶90.9
**Redcurrant leaves**	24.5	6.0∶ 94.0	9.8	77.3∶22.7
**White clover flowers**	33.8	0.8∶ 99.2	4.4	65.7∶34.3

CT = condensed tannins, PC = procyanidin, PD = prodelphinidin, mDP = mean degree of polymerization.

**Figure 2:**
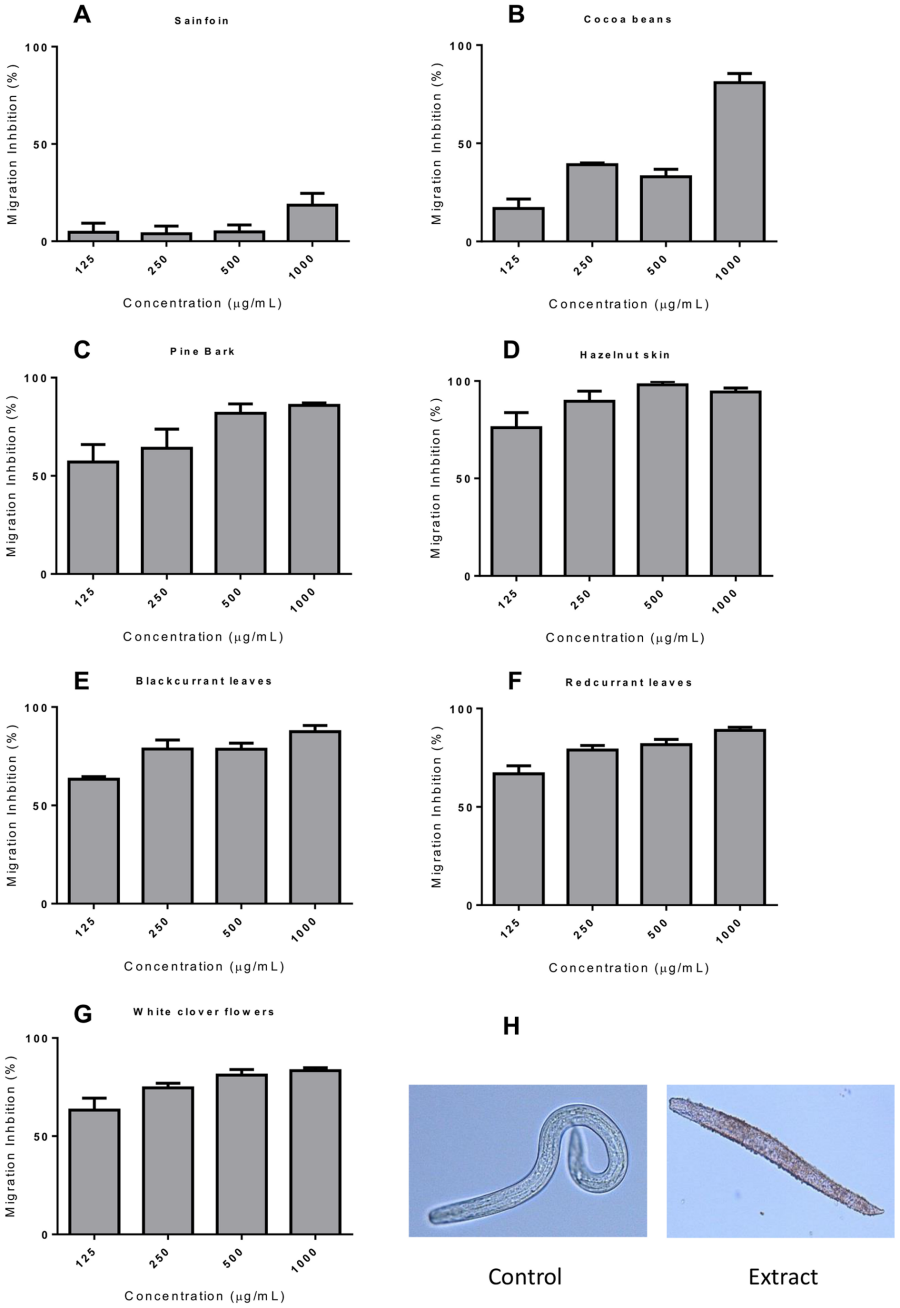
Inhibition of *Ascaris suum* L3 migration by acetone/water extracts of tannin-containing plants. Panel A–G - Percentage inhibition of migratory activity (MIA) in *Ascaris suum* L3 after 16 hours exposure to tannin-containing acetone/water extracts from **A)** sainfoin, **B)** cocoa beans, **C)** pine bark, **D)** hazelnut skins, **E)** blackcurrant leaves, **F)** redcurrant leaves and, **G)** white clover flowers. MIA is expressed relative to larvae exposed only to culture medium. Data presented are the mean of two independent experiments, each performed in triplicate. Error bars represent SEM of the individual replicates. **Panel H –** Light microscopy of *A. suum* larvae after migration following incubation in media only, or after migration following incubation in 1 mg/mL of tannin-containing acetone/water extract from pine bark (magnification = x200).

After ingestion of eggs and hatching *in vivo*, *A. suum* L3 must then undergo a migratory phase which consists of migration through the intestinal wall and through to the liver to the lungs, before they return to the small intestine. Therefore, we also investigated whether the migratory ability of *A. suum* L3 was impaired after incubation with the extracts by using an agar migration inhibition assay. In the negative control wells, a mean of 67% of larvae (SEM ±7.2, range 58–82) of larvae were able to migrate out of the agar gel, and this migration was inhibited by a mean of 86% (SEM ±4.4, range 73–99) by addition of 50 µg/mL ivermectin to the media at the start of the incubation period. We observed potent, dose-dependent MIA in larvae exposed to six out of seven extracts – in agreement with the motility data, larvae exposed to sainfoin did not have significantly reduced migration (Figure 2). The MIA was similar whether the agar was added directly to the wells containing media and tannin extracts after the overnight incubation, or whether the incubated larvae were first washed and then re-suspended in fresh media without tannins before the addition of agar (Figure S3), indicating that the MIA was mediated by direct effects of the extracts on the larvae during the incubation period, and not by non-specific inhibition caused by interactions between tannins and agar influencing the composition and penetrability of the agar gel. Moreover, larvae that did migrate from the gel after incubation in tannin-containing extracts appeared sluggish and were often dead at high concentrations of extract (Figure 2H), indicating that they had died after migrating but before subsequent observation by microscopy. Altogether, these data suggest that extracts of plants containing high amounts of CT have direct anthelmintic activity against *A. suum*.

### Condensed Tannins are Mainly Responsible for Anthelmintic Activity against *A. suum*

**Figure 3:**
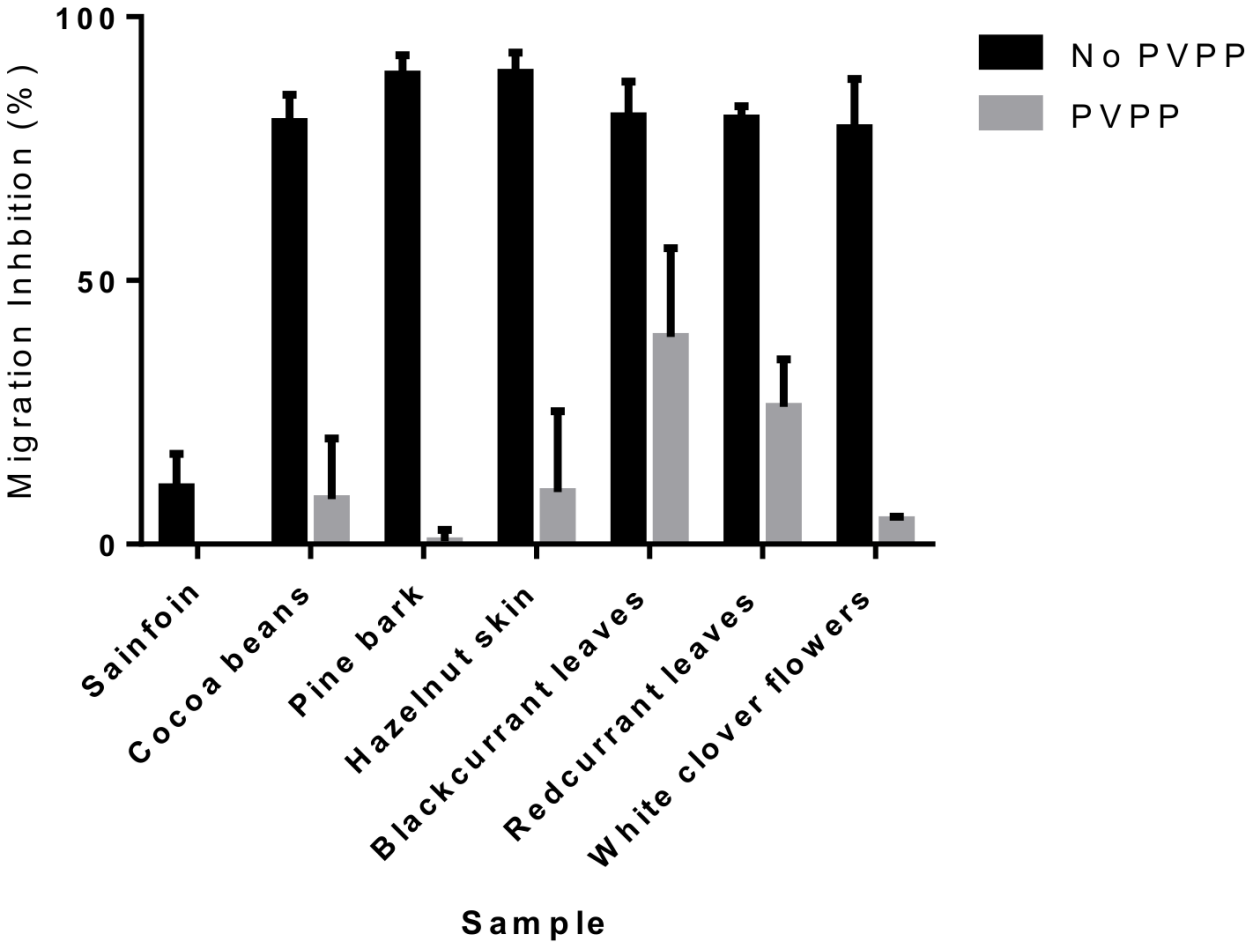
Effect of PVPP treatment on anthelmintic activity of tannin-containing plant extracts. Percentage inhibition of migratory activity (MIA) in *Ascaris suum* L3 after 16 hours exposure to tannin-containing acetone/water extracts from sainfoin, cocoa beans, pine bark, hazelnut skins, blackcurrant leaves, redcurrant leaves and white clover flowers, or the same extracts after pre-incubation with PVPP (see materials and methods). MIA is expressed relative to larvae exposed only to culture medium (no PVPP), or culture medium pre-incubated with PVPP (PVPP). Data presented is the mean of two independent experiments, each performed in triplicate. Error bars represent SEM of the individual replicates.

We noted that the extracts that had the most potent activity against *A. suum* were those extracts that had the highest levels of CT. For example, the extract from hazelnut skin contained 70% CT, whereas the extracts that exhibited the least activity (cocoa and sainfoin) had the lowest percentage of CT (Table 1 and Figure 2). This suggests that CT are the active compounds responsible for the observed anthelmintic activity. To confirm this, we pre-treated the extracts with PVPP, which selectively binds and precipitate tannins [31]. With the exception of sainfoin, where only minimal MIA was observed, pre-treatment with PVPP significantly reduced the anthelmintic effect in all of the extracts (*P*<0.001 by two-way ANOVA; Figure 3), although with different levels of entirety. PVPP treatment almost completely abolished the effect in extracts of cocoa beans, pine bark, hazelnut skin and white clover flowers, however this reduction was less apparent in extracts from redcurrant and blackcurrant leaves, indicating that either removal of tannins by PVPP was incomplete in these cases or there are small amounts of other, unidentified bioactive substances in these extracts. Overall, these results strongly suggest that CT are mainly responsible for the observed anthelmintic effects.

### Fractionation of Extracts and Further Purification of Condensed Tannins

To further confirm the role of CT in the anthelmintic effects, and to gain insight into the relationship between tannin structure and activity, we fractionated and purified the extracts to yield two tannin fractions. The first fraction (F1) yielded oligomeric tannins (mDP of 2 to 4) with around 50% CT content, whereas the second fraction (F2) contained mainly oligomers and polymers of higher molecular weight tannins (mDP>6) and close to 100% CT content (Table 2).

**Table 2:** Chemical analysis of fractionated tannins.

Sample		CT (g/100 g fraction)	PC : PD ratio	mDP	*cis* : *trans* ratio
**Sainfoin**	F1	37.8	28.0∶ 72.0	2.7	66.7∶33.3
	F2	100.0	35.2∶64.8	8.7	79.2∶20.8
**Cocoa beans**	F1	58.5	100.0∶0.0	2.3	91.3∶ 8.7
	F2	75.5	100.0∶0.0	5.4	96.3∶ 3.7
**Pine bark**	F1	56.5	84.9∶15.1	2.3	48.1∶ 51.9
	F2	83.8	88.8∶11.2	6.6	78.1∶ 21.9
**Hazelnut skin**	F1	51.3	81.7∶ 18.3	4.6	41.0∶ 59.0
	F2	70.3	79.1∶20.9	9.1	47.8∶ 52.2
**Blackcurrant leaves**	F1	59.9	6.3∶ 93.7	2.5	12.8∶ 87.2
	F2	100.0	5.5∶94.5	6.5	7.0∶93.0
**Redcurrant leaves**	F1	58.1	14.2∶85.8	4.9	44.4∶ 55.6
	F2	68.5	9.7∶90.3	10.0	64.4∶35.6
**White clover flowers**	F2	100.0	1.3∶98.7	8.6	58.9∶ 41.1

Plant extracts were fractionated on Spehadex-LH20 columns yielding fraction 1 (F1) and fraction 2 (F2). CT = condensed tannins, PC = procyanidin, PD = prodelphinidin, mDP = mean degree of polymerization.

**Figure 4:**
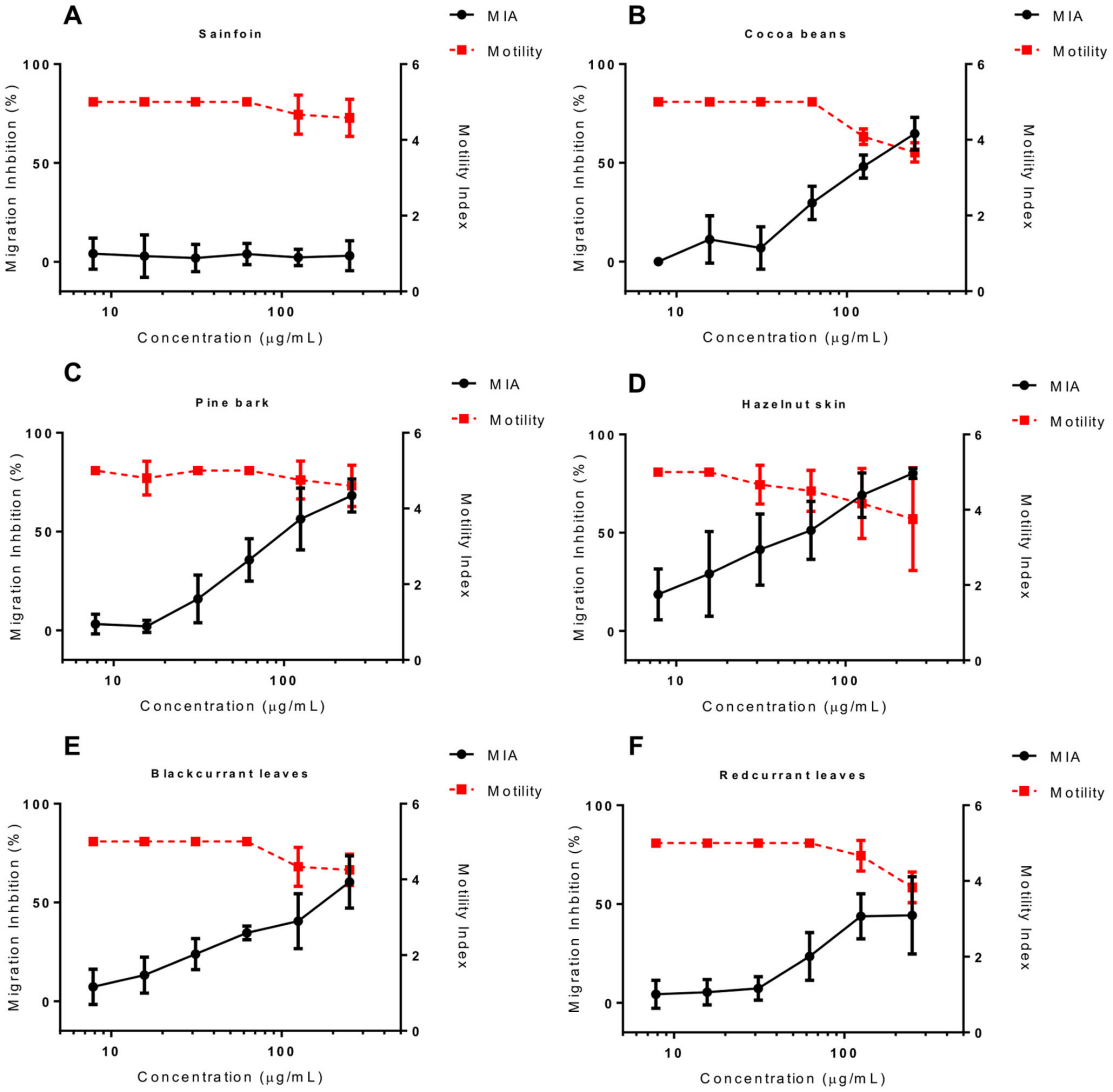
Inhibition of *Ascaris suum* L3 motility and migration by condensed tannin fractions (F1). Percentage inhibition of migratory activity (MIA - solid black line, plotted on left Y axis) and motility (dashed red line, plotted on right Y axis) in *Ascaris suum* L3 after 16 hours exposure to tannin fractions (F1) from **A)** sainfoin, **B)** cocoa beans, **C)** pine bark, **D)** hazelnut skins, **E)** blackcurrant leaves and **F)** redcurrant leaves. MIA is expressed relative to larvae exposed only to culture medium. Motility is scored on a 0–5 scale where 5 is completely motile and 0 is completely still (see materials and methods). Data points represent the mean of two independent experiments, each performed in triplicate. Error bars represent SEM of the individual replicates.

**Figure 5:**
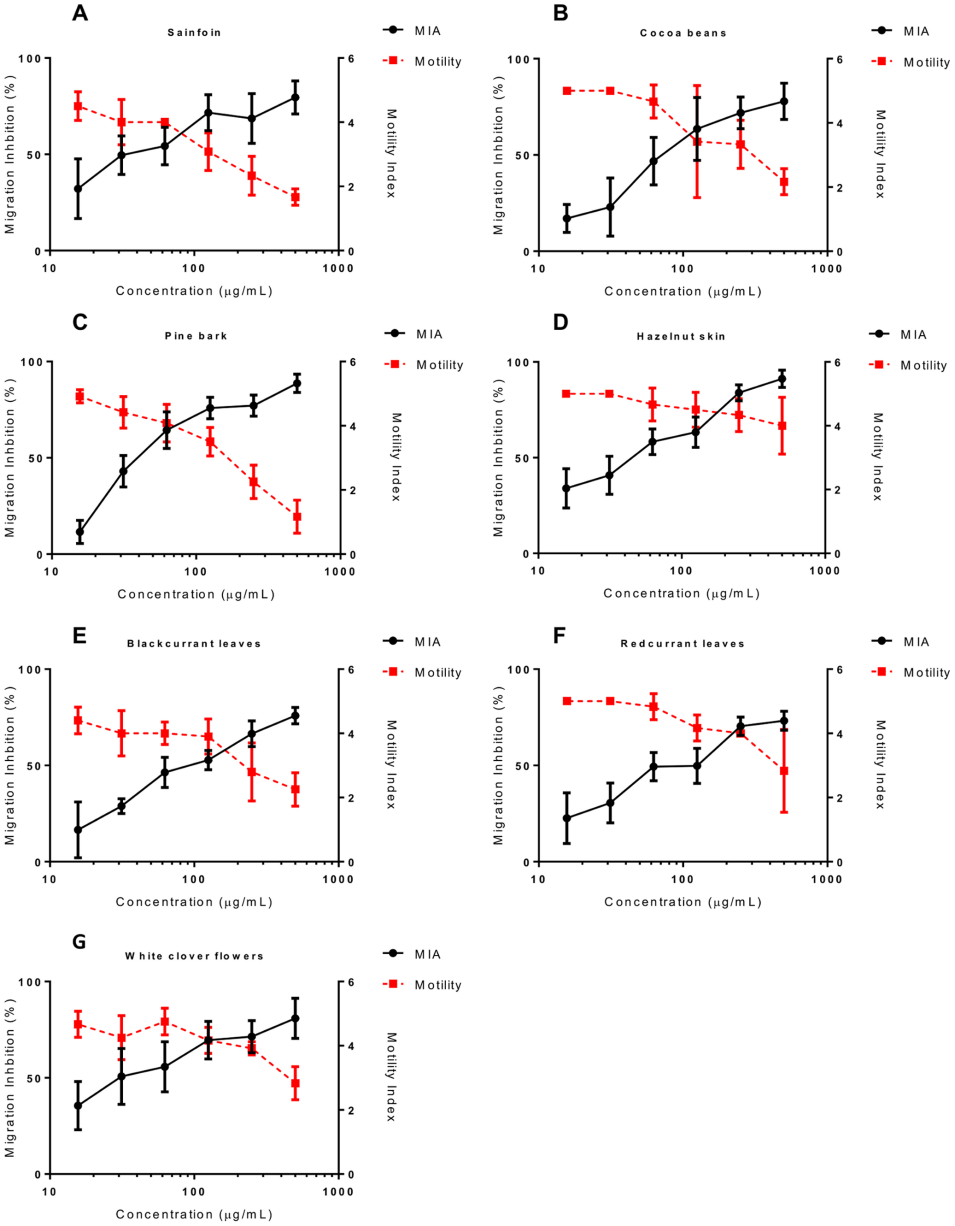
Inhibition of *Ascaris suum* L3 motility and migration by condensed tannin fractions (F2). Percentage inhibition of migratory activity (MIA - solid black line, plotted on left Y axis) and motility (dashed red line, plotted on right Y axis) in *Ascaris suum* L3 after 16 hours exposure to tannin fractions (F2) from **A)** sainfoin, **B)** cocoa beans, **C)** pine bark, **D)** hazelnut skins, **E)** blackcurrant leaves, **F)** redcurrant leaves and **G)** white clover flowers. MIA is expressed relative to larvae exposed only to culture medium. Motility is scored on a 0–5 scale where 5 is completely motile and 0 is completely still (see materials and methods). Data points represent the mean of two independent experiments, each performed in triplicate. Error bars represent SEM of the individual replicates.

We then assessed the motility and MIA in larvae exposed to varying concentrations of these fractions. To allow comparison of different tannin samples, the concentrations of the fractions were adjusted in the assay so that all F1 samples contained the same amount of CT (final concentrations ranging from 250 to 7.8 µg/mL), and similarly the F2 samples were adjusted so that all contained CT of concentrations ranging from 500 to 15.6 µg/mL. Similar to the previous results with the crude acetone/water extract, the F1 fraction of sainfoin did not show any anthelmintic activity (Figure 4A). However, F1 fractions from cocoa, hazelnut skin, pine bark and blackcurrant and redcurrant leaves all showed a modest effect on larval motility, and a more pronounced effect on MIA (Figure 4B–F). EC_50_ values were calculated for MIA, with CT from pine bark and hazelnut skin being the most potent (Table 3). The F2 fractions showed more potent anthelmintic activity, with a stronger effect on larval motility and MIA (Figure 5). Interestingly, F2 fractions from sainfoin (which had shown no anthelmintic activity when tested either as a crude acetone/water extract or a low molecular weight F1 fraction), were now highly active with potent effects on both larval motility and migratory ability (Figure 5A). EC_50_ values for MIA were (with the exception of hazelnut skin, where values were similar between fractions) significantly lower for the F2 samples than for the corresponding F1 fraction (Table 3; *P*<0.01 by extra sum-of-squares F-test), indicating that CT from the second fraction were more effective at inhibiting the migration of *A. suum* than an equivalent concentration of CT from the first fraction. This suggests that the polymer size of the tannin molecules is an important factor in determining their anthelmintic activity, with a higher degree of polymerization increasing the efficacy. However, within F2 fractions from different plant sources there was no effect of mDP on MIA EC_50_, nor was there any effect of the PC/PD or the *cis*/*trans* ratios (*P*>0.1 by multiple regression).

**Table 3:** *Ascaris suum* L3 migratory inhibition activity EC_50_ values.

Sample	Sainfoin	Cocoa beans	Pine bark	Hazelnut skin	Blackcurrant leaves	Redcurrant leaves	White clover flowers
**Fraction 1**	NC	141.3	114.8	49.6	162.0	247.9	ND
**Fraction 2**	41.9	86.0	48.2	43.8	98.4	91.8	37.4

Inhibition of migration was assessed in L3 exposed to purified tannins extracted from sainfoin, cocoa beans, pine bark, hazelnut skin, blackcurrant leaves, redcurrant leaves and white clover flowers. ND = not done. NC = not calculated. For all samples except hazelnut skins, the EC_50_ values are significantly lower for the F2 fraction compared to the F1 fraction (*P*<0.01 by extra sum-of-squares F-test).

### Flavanol Monomers of Prodelphinidins have Higher Larval Migratory Inhibitory Activity than Monomers of Procyanidins

**Figure 6:**
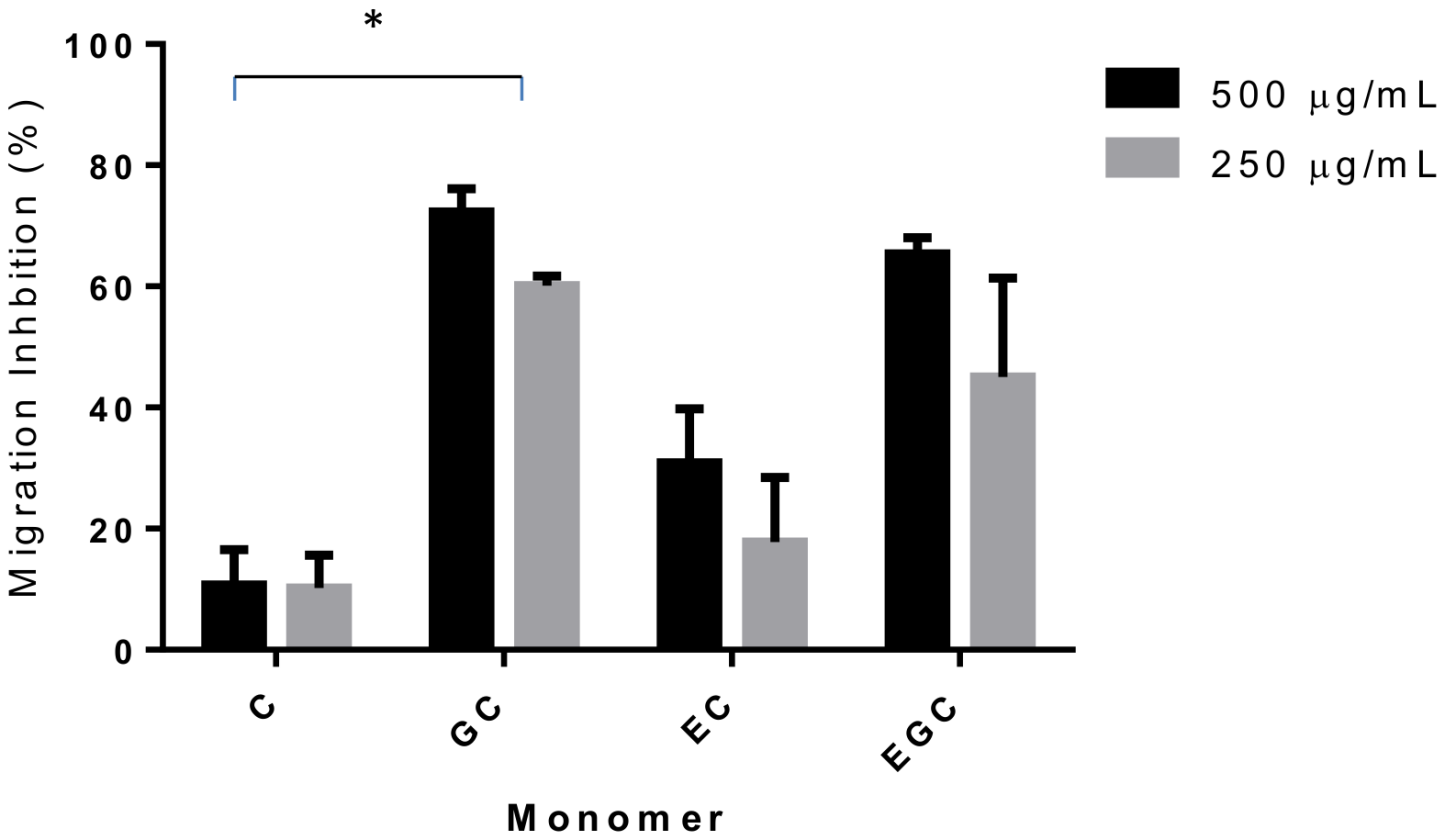
Inhbition of *Ascaris suum* L3 migration by flavanol monomers. Percentage inhibition of migratory activity (MIA) in *Ascaris suum* L3 after 16 hours exposure to flavanol monomers: catechin (C), gallocatechin (GC), epicatechin (EC) and epigallocatechin (EGC). Data points represent the mean of two independent experiments, each performed in triplicate. Error bars represent SEM of the individual replicates. *These values differ significantly (P<0.01, two-way ANOVA with Bonferroni post-hoc testing) at both concentrations (500 and 250 µg/mL).

Previous studies of the effects of CT on ruminant nematodes have shown that the ratio of prodelphinidins (PD) to procyanidins (PC) in the CT polymer and their corresponding flavanol monomers influence the anthelmintic activity, with a high percentage of PD thought to increase the potency of anthelmintic effects [22], [32]. However, multiple regression analysis of our current data with purified F2 fractions showed no correlation between the % of PD units and MIA EC_50_ with L3. The multitude of different tannin molecules, which are present even in purified fractions, may make it difficult to identify structural characteristics that influence activity. Therefore, we also tested commercially available flavanol monomers (catechin (C), epicatechin (EC), gallocatechin (GC) and epigallocatechin (EGC)) to address whether PD/PC ratio and also *cis*/*trans* ratio may be important in the observed anthelmintic effects. We observed that GC and EGC, which occur in prodelphinidins, both reduced motility of newly hatched L3 (Figure S4), and also markedly inhibited L3 migration at concentrations of 500 and 250 µg/mL (Figure 6). In contrast, larval motility was not significantly reduced by incubation with C or EC, which occur in procyanidins, and the percentage of MIA was lower than for the corresponding GC and EGC flavanols (Figure S4 and Figure 6). Analysis of variance revealed that MIA was significantly different between the GC and C monomers (*P*<0.01 by two-way ANOVA), however the difference between EGC and EC did not reach significance, nor was there any significant effect of *cis*/*trans* stereochemistry. These data suggest that the presence of an extra hydroxyl group in the B-ring of the flavanols increases the anthelmintic activity, and are in agreement with previous studies that investigated the anthelmintic effects of these flavanols against *H. contortus* [22], [32]. However, our results with purified mixtures of CT oligomers and polymers appear to indicate that, in these cases, the PD/PC ratio is less important or is masked by other factors.

### Condensed Tannins also have Anthelmintic Activity against Ascaris Suum Fourth-stage Larvae

Whilst the L3 migration assay provides a convenient means by which to screen for anthelmintic activity, it is perhaps more relevant to the *in vivo* situation to examine effects against the L4 stage of the parasite. After the L3 have migrated through the liver to the lungs, they are coughed up and return to the small intestine around 8 days post-infection (p.i.), and at around day 10 p.i. they moult to the L4 stage [33]. The parasite then resides in the small intestine up until adulthood and for the remainder of its lifespan, and is thus exposed for long periods to compounds present in the host digesta. Thus, it may be this post-migration stage of the parasite life-cycle that is most susceptible to anthelmintic dietary compounds. Therefore, we also examined the effects of acetone/water extracts and corresponding F2 CT fractions from sainfoin, cocoa beans, pine bark, hazelnut skin, blackcurrant leaves and white clover flowers on the motility and survival of *A. suum* L4 compared to culture media with no tannins.

**Figure 7:**
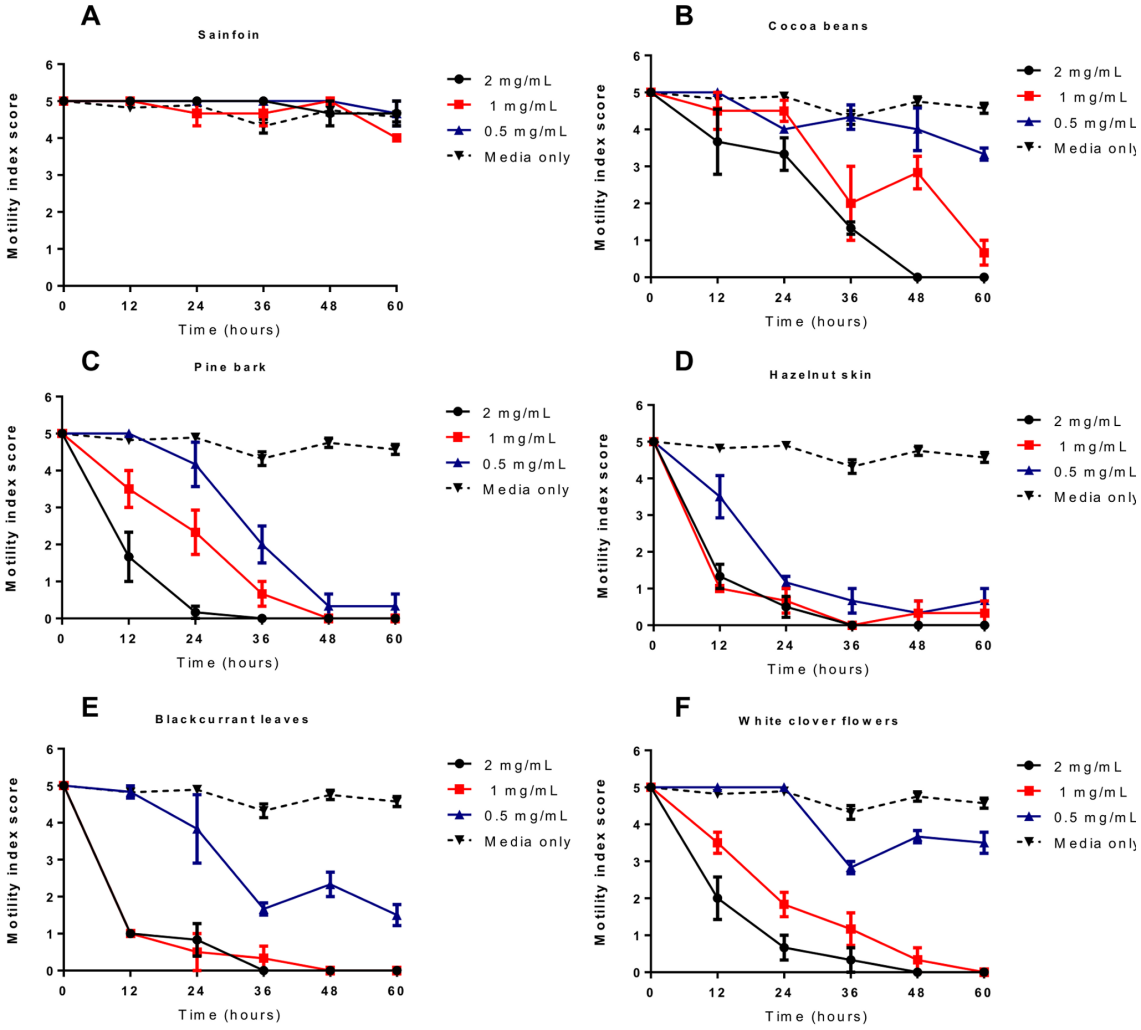
Inhibition of *Ascaris suum* L4 motility by acetone/water extracts of tannin-containing plants. Motility of *Ascaris suum* L4 exposed to tannin-containing acetone/water extracts of **A)** sainfoin, **B)** cocoa beans, **C)** pine bark, **D)** hazelnut skins, **E)** blackcurrant leaves and **F)** white clover flowers. Data points represent the mean of triplicate wells, with the error bars representing the inter-well SEM.

**Figure 8:**
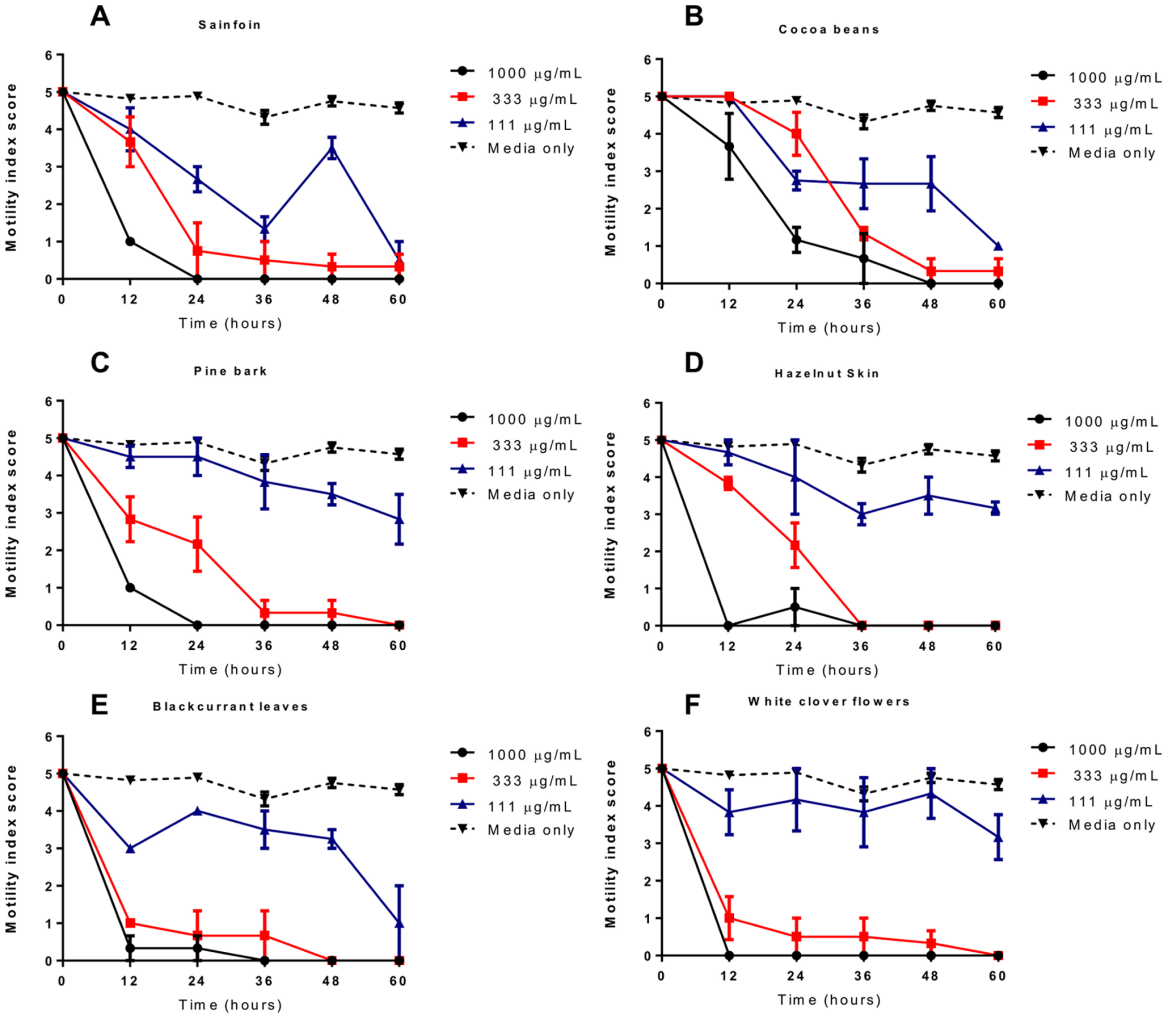
Inhibition of *Ascaris suum* L4 motility by condensed tannin fractions (F2). Motility of *Ascaris suum* L4 exposed to varying concentrations of tannins (F2 fractions) from **A)** sainfoin, **B)** cocoa beans, **C)** pine bark, **D)** hazelnut skins, **E)** blackcurrant leaves and **F)** white clover flowers. Data points represent the mean of triplicate wells, with the error bars representing the inter-well SEM.

Parasites were recovered from pigs 12 days p.i., with >80% of the recovered larvae determined to be L4 by morphological examination [33], with the remainder being late L3. Larvae incubated in media alone remained fully motile over the course of the 60 hours of incubation. In contrast, larvae exposed to either ivermectin or levamisole had a significant reduction in motility within 12 hours, which persisted to the end of the experiment (Figure SF5). Larvae exposed to the sainfoin acetone/water extract did not show significantly reduced motility at any concentration tested, consistent with the poor anthelmintic activity observed in the previous assays (Figure 7A). In contrast, larvae exposed to the acetone/water extracts from the other five plant sources all had lower motility than controls, with an almost total cessation of movement at concentrations of ≥1 mg/mL by the end of the experiment (Figure 7B–F). Results with the F2 fractions confirmed the anthelmintic effects. Larvae exposed to all tested fractions exhibited a rapid reduction in motility (Figure 8), most notably in the prodelphinidin-rich white clover and blackcurrant leaf fractions where larvae exposed to either 1000 or 333 µg/mL of CT exhibited almost a total loss of movement within 12–24 hours (Figure 8E–F). In all tested fractions, all larvae exposed to 1 mg/mL of tannins were dead by the end of the experiment, and reductions in motility were also observed in the lowest concentration of 111 µg/mL (Figure 8). These results indicate that CT have potent anthelmintic effects against the L4 stage as well as the L3 stage of *A. suum.*

### Ultrastructural Changes in Larvae Exposed to Condensed Tannins

**Figure 9:**
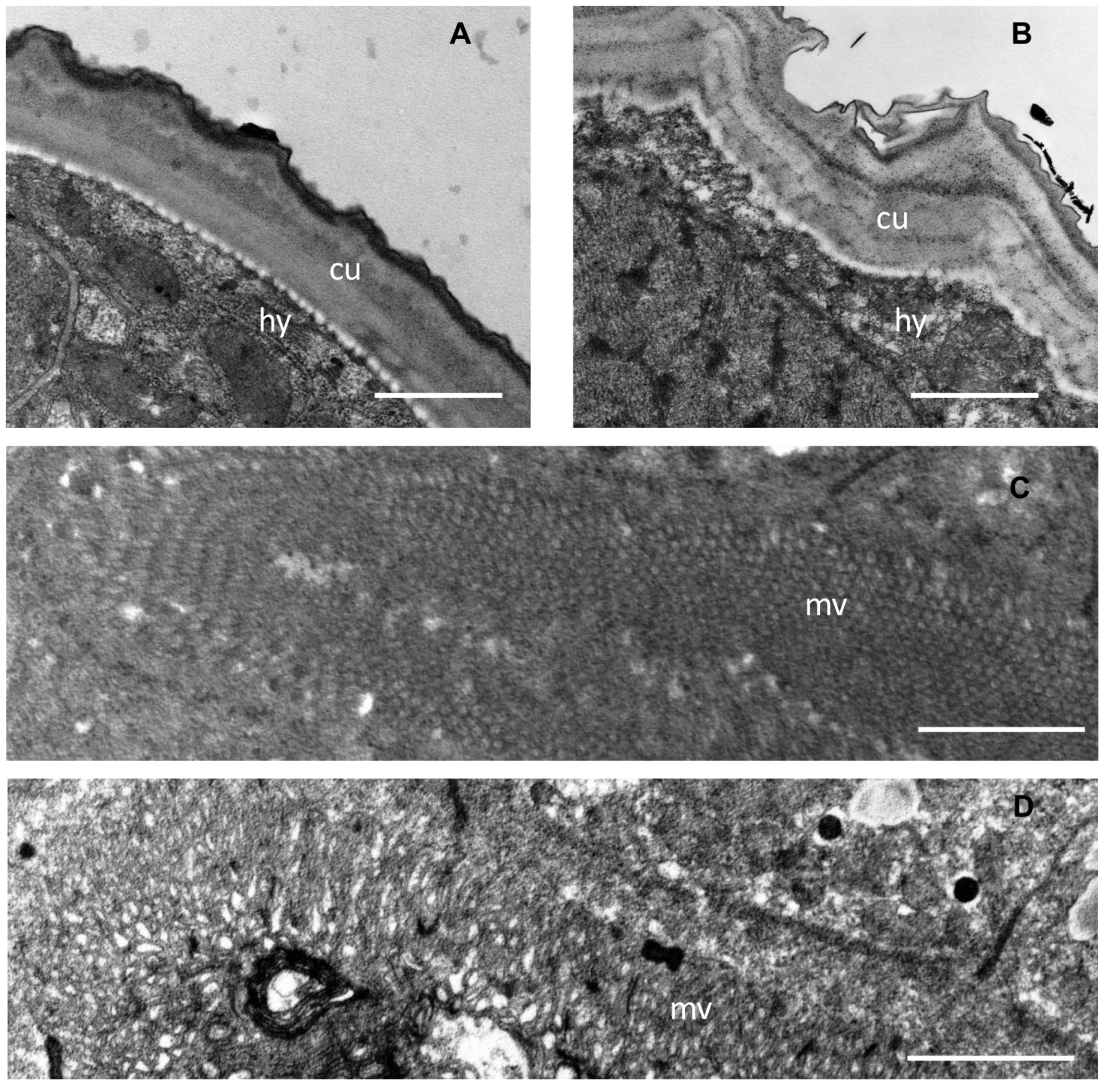
Ultrastructural changes in *Ascaris suum* L4 exposed to condensed tannins. Transmission electron microscopy of thin sections through *Ascaris suum* larvae recovered from pigs 12 days post-infection and then incubated for 24 hours in either culture media alone (**A,C**) or with 1 mg/mL condensed tannins (fraction 2 from hazelnut skin – **B,D**). **Panel A, B** – ‘cu’ indicates cuticle, ‘hy’ indicates hypodermis; scale bar represents 1 µm. **Panel C,D** – ‘mv’ indicates microvilli in brush-border of intestine; scale bar represents 2 µm.

Transmission electron microscopy (TEM) was performed to investigate possible direct structural damage to L4 exposed to CT. Larvae recovered from pigs and incubated in 1 mg/mL of CT from hazelnut skin were motionless after 12–24 hours; hence worms from this treatment were selected for analysis by TEM. Thin-sections prepared from larvae after 24 hours of incubation revealed significant structural damage (Figure 9). The cuticle was noticeably swollen and irregular, with marked disorganisation of the basal layer and the underlying hypodermis, compared to control larvae which displayed an intact and regular shaped cuticle (Figure 9A–B). Moreover, massive damage was also observed in the intestine with marked destruction of microvilli in the brush-border and the presence of vacuoles in the gut tissue (Figure 9C–D), confirming direct structural damage to both external and internal tissues of worms exposed to CT.

## Discussion

The use of plant compounds such as CT as anthelmintics has received renewed interest due to the on-going threat of parasite resistance to the small number of synthetic drug classes currently available, as well as concerns about drug residues arising from prophylactic use of synthetic drugs in food production systems. Whilst the potential of CT to be used as anthelmintics has been repeatedly demonstrated in parasites from ruminant livestock, as well as in some rodent models, evidence of direct anthelmintic effects against the pig parasite *A. suum* has been lacking. While some studies have hinted at possible anthelmintic effects [34],[35], our results show clearly that multiple larval stages of *A. suum* are susceptible to purified CT derived from a wide variety of plant sources. This provides clear evidence of direct *in vitro* effects against this parasite and encourages further *in vivo* investigation of the use of CT for control of *A. suum* as well as the closely related *A. lumbricoides.*

With the somewhat surprising exception of sainfoin, acetone/water extracts of all the tested plant samples strongly inhibited the migratory ability of newly hatched *A. suum* larvae. Whilst these crude extracts contain other compounds besides CT, pre-treatment with PVPP significantly reduced the anthelmintic effect and abolished it in many cases, strongly implicating CT as the active molecules, a result further confirmed as purified fractions containing near to 100% CT retained potent anthelmintic effects. These results are consistent with studies with other parasitic nematodes such as *H. contortus*, where reduced ability of larvae to migrate has been repeatedly observed after contact with CT. Intestinal-dwelling parasites such as *A. suum* need to retain motility and muscular co-ordination in order to avoid being removed from the gut by peristalsis. Indeed, the mode-of-action of many synthetic drugs such as macrocyclic lactones (e.g. ivermectin) is to remove this motility and co-ordination by activation of glutamate-gated chloride channels [36], resulting in paralysis and removal from the intestine by contractions of normal gut function. Therefore, although the L3 were mostly still alive after overnight incubation with CT, their viability was compromised, as evidenced by their inability to migrate from agar gel. This agar migratory assay is a rather stringent test of the viability of the larvae, as it requires sustained, active migration in a vertical direction and probably resembles the type of environment that larvae may find themselves in *in vivo,* where they have to traverse semi-solid matrices such as mucus and intestinal tissue to begin the hepatic-tracheal migration. Our data suggest that the presence of CT in the digesta will compromise the ability of larvae to undergo this migration out of the intestine, which takes place in the caecum and upper colon. Moreover, the L4 stage, which is present in the small intestine after this migration and exposed for a lengthy period to the host digesta, was also potently affected by CT with significant larval death occurring in the L4 motility assay. Whilst we did not investigate effects on adult worms in these experiments, other studies with parasitic nematodes have demonstrated a significant reduction in the egg-laying ability of adult females exposed to CT [17]. Overall, these results suggest that CT have anthelmintic activity towards multiple stages of the *Ascaris* life-cycle.

These results should allow rational selection of natural plant extracts that may aid in the treatment and/or prevention of *Ascaris* infections. Whilst the use of plant extracts to treat helminth infections in both pigs and humans is widespread in developing countries, a lack of scientific validation of the active compounds of such plants has hampered the optimisation of these treatment protocols. Condensed tannins are commonly found in plant sources worldwide, and may be administered in several ways. First, livestock may be directly fed whole foodstuffs or forages containing CT. Second, aqueous or alcoholic extracts may be prepared from plant material such as bark or roots and administered as food supplements or in medicinal form. Finally, CT are commonly present in agricultural by-products such as cocoa husks, hazelnut skins or coffee pulp. Such waste products may represent inexpensive sources of CT for many small-holder pig production systems in developing countries. Moreover, the use of CT-containing forages and/or by-products may represent a sustainable method for *A. suum* control in low-input and organic pig farms in developed countries where the routine use of synthetic anthelmintic drugs is not allowed. The use of CT as part of an integrated parasite control program will have the benefit of reducing reliance on the small number of currently available synthetic drugs, slowing the threat of drug-resistant parasites and reducing reliance on expensive drugs, as well as meeting consumer demand for food products produced with minimal input of synthetic antimicrobials.

The extrapolation of these *in vitro* results to the *in vivo* situation requires several considerations. Perhaps the most important consideration is what concentration of CT is required to be administered to achieve *in vivo* efficacy. We have, as far as possible, attempted to use concentrations of CT in our *in vitro* assays that represent physiological concentrations that may be expected to be found in the intestine of pigs ingesting CT, although such information in the scientific literature is scant. However, several assumptions can be drawn from studies where humans, pigs and other monogastric animals have consumed diets containing CT. It has been repeatedly shown that the absorption of oligomeric CT from the intestine of monogastrics (including humans and pigs) is poor, with only trace amounts, mainly of monomers or dimers and their metabolites, being detected in the plasma or urine after ingestion. Indeed, much of the orally administered CT can be recovered from digesta at the terminal ileum, suggesting that there is minimal absorption of CT along the small intestine [12], [37]–[40]. This stability of oligomeric CT through the stomach and proximal part of the small intestine implies that parasites such as *A. suum,* whose L4 and adult stages reside in the small intestine should come into contact with CT at sufficiently high concentrations to exert biological activity, such as has been recently reported for pigs infected with *Escherichia coli*, whereby feeding CT-enriched extracts significantly reduced *E. coli* colonisation of the intestine [41]. Moreover, this poor absorption of CT has generally led them to being considered to have low toxicity; however detrimental effects may be observed in the form of reduced nutrient absorption due to formation of insoluble complexes with CT, as well as the possibility of irritation of the gastrointestinal mucosa at high concentrations [12]. Therefore, it would appear that there is an upper limit to the amount of CT that can be ingested without side-effects; however it is likely that anthelmintic effects will be manifested at concentrations well below this threshold. In pigs, the volume of the digesta in the small intestine of a growing animal (40–60 kg) is around 5L, depending on age [42]. Thus, to achieve a concentration of 1 mg/mL of CT in the small intestine it will require the animal to ingest around 5 g of CT. As a growing pig most likely eats around 2 kg of food a day, this level of CT represents less than 0.25% of the daily dietary intake. In practice, the ingested amount will need to be higher, in order to compensate for some break-down and decreased bioavailability due to binding to dietary or endogenous proteins and fibres, however it should be still well within the limits of what can be feasibly incorporated into the diet with no adverse effects. Indeed, some *in vivo* verification is already in existence in a monogastric model; burdens of the rodent parasite *Nippostronglyus brasilleinas*, another parasitic nematode which resides in the small intestine, were significantly reduced in rats fed a diet of 4% CT [18]. Moreover, given that *A. suum* infection may impose a significant penalty on the nutrient economy of the animal [2], the benefits of CT intake may outweigh any possible anti-nutritional effects. Therefore, ingestion of dietary supplements or extracts containing CT should feasibly exert *in vivo* anthelmintic activity, although further experiments are required to substantiate this, which forms part of on-going work in our laboratory.

The lack of effect with the acetone/water extract or F1 fraction of sainfoin, despite potent anthelmintic activity with the F2 fraction is not easily explained. The most logical postulate is that impurities in the extract and F1 fraction interfere with the CT, and that these impurities are removed in the pure F2 fraction. However, it is not clear what such impurities may be. Sainfoin contains a large amount of sucrose [43], however pre-treatment of the F2 fraction with sucrose did not remove its anthelmintic activity (data not shown). Moreover, previous studies have demonstrated marked *in vitro* anthelmintic effects of both aqueous extracts and associated CT fractions of sainfoin towards other parasitic nematodes [22], [44], [45], although it cannot be ruled out that this phenomenon is specific to *A. suum*, or else to the particular accession of sainfoin used in our current study. Further studies will be necessary to elucidate this; for now, our results highlight the importance of considering the effects of complex extracts from plants, as well as pure compounds, when the whole plant or crude extract is to be used as a potential anthelmintic agent.

We observed significantly lower EC_50_ values in the L3 migration assay for the F2 tannin fractions than for the corresponding F1 fraction for each sample, with the exception of hazelnut skin. Of note, the F1 fraction from hazelnut skin had the highest mDP (4.1) of all the F1 fractions (Table 2) – perhaps explaining the lack of significant difference in MIA between the F1 and F2 fractions for this sample, as the size of the F1 fraction was still sufficient to exert significant anthelmintic activity. We have also previously noted a correlation between the mDP of CT extracted from sainfoin and the degree of anthelmintic activity against the cattle parasitic nematodes *Cooperioa oncophra* and *Ostertagia ostertagi* [45]. The reasons for this relationship are not yet clear. It is thought that CT may exert anthelmintic activity by binding to proline-rich proteins on the cuticle or in the digestive system [32], and indeed we observed evidence of significant damage to these tissue in thin sections of larvae exposed to CT. It may be that the binding of larger CT polymers exerts a steric hindering effect on neighbouring parasite molecules. Protein-CT binding experiments have shown that CT coat the surface of proteins such as bovine serum albumin (M. Dobreva and IMH, manuscript in preparation), and it is conceivable that such a coating effect will interfere with the function of key parasite proteins and free movement of larvae, similar to the role proposed for host antibodies in growth inhibition of *A. suum* larvae [46]. Further experiments are necessary to elucidate these mechanisms. However, it is apparent that while CT size may contribute to the activity, anthelmintic effects can still be mediated by smaller CT molecules; indeed, significant activity was observed with the GC/EGC monomers (but not the C/EC monomers). The increased activity of GC/EGC monomers has previously been noted with experiments with *H. contortus* and *Trichostrongylus colubriformis* [32], [47], as well as with other microbes such as *Streptococcus* [48]. The increased hydroxylation in the B-ring of PD monomers is thought to favour increased hydrogen-bonding with proteins and thus increase biological activity. However, despite this clear effect with monomers, we were unable to detect a strong influence of PD/PC ratio in the activity of the purified tannins – whilst CT purified from white clover flowers (made up>98% of PD units) appeared to have the most potent anthelmintic activity (Table 3 and Figure 8) – CT purified from blackcurrant and redcurrant leaves, which are also comprised almost exclusively of PD had similar activity in the MIA assay to cocoa and pine bark, both of which are comprised of 85–100% PC units.

In conclusion, we have provided clear evidence of the direct anthelmintic effects of CT against *Ascaris suum*. We have also highlighted several structural features of CT which may influence the anthelmintic activity. These studies pave the way for further, *in vivo* studies to determine the optimal use of CT as a sustainable and complementary means of control of *Ascaris suum* in pigs and *Ascaris lumbricoides* in humans.

## Supporting Information

Figure S1 **Explanation of thiolytic degradation scheme for determining chemical composition of condensed tannin molecules.**
(TIF)

Figure S2 **Motility of** ***Ascaris suum*** **L3 after exposure to extracts from tannin-containing plants.** Motility of *Ascaris suum* L3 after 16 hours exposure to tannin-containing acetone/water extracts from hazelnut skins (HN), cocoa (COC), pine tree bark (PTB), sainfoin (SF), blackcurrant leaves (BC), redcurrant (RC) leaves and white clover flowers (WC). Motility is scored on a 0–5 scale where 5 is completely motile and 0 is completely still (see materials and methods). Dashed black line indicates the motility of larvae exposed to only culture medium (negative control). IVM = ivermectin at 50 µg/mL (positive control). Data points represent the mean of two independent experiments, each performed in triplicate. Error bars represent SEM of the individual replicates.
(TIF)

Figure S3 **Effect of washing on inhibition of migratory activity (MIA).** *Ascaris suum* L3 were incubated for 16 hours in tannin-containing acetone/water extracts from hazelnut skins, redcurrant leaves or pine bark. Larvae were then washed to remove tannins and resuspend in fresh media before addition of agar (‘washing’), or agar was added directly to the larvae in the tannin-containing media (‘no washing’).
(TIF)

Figure S4 **Motility of** ***Ascaris suum*** **L3 after exposure to flavanol monomers.** Larvae were incubated for 16 hours with either catechin (C), gallocatechin (GC), epicatechin (EC) or epigallocatechin (EGC). Motility is scored on a 0–5 scale where 5 is completely motile and 0 is completely still (see materials and methods). Data points represent the mean of two independent experiments, each performed in triplicate. Error bars represent SEM of the individual replicates.
(TIF)

Figure S5 **Motility of** ***Ascaris suum*** **L4 exposed to synthetic anthelmintic drugs.** Motility of *Ascaris suum* L4 exposed to either 100 µg/mL ivermectin (IVM) or levamisole (LVM), or culture media only. Data points represent the mean of triplicate wells, with the error bars representing the inter-well SEM.
(TIF)

Checklist S1 **ARRIVE Checklist.**
(DOC)

Table S1 **Proportions of monomeric flavanol subunits in extracts and fractions.** Proportions of gallocatechin (GC), epigallocatechin (EGC), catechin (C) and epicatechin (EC)
(DOCX)
