# Correction: Direct anthelmintic effects of condensed tannins from diverse plant sources against *Ascaris suum*

**DOI:** 10.1371/journal.pone.0099738

**Published:** 2014-06-04

**Authors:** The PLOS ONE Staff

The Data Availability statement does not appear on the paper. The correct Data Availability statement is: The authors confirm that all data underlying the findings are freely available without restriction. All data are included within the paper and its Supporting Information files.

The publisher apologizes for this error.
