# Supplementary figures and images for: Direct Anthelmintic Effects of Condensed Tannins from Diverse Plant Sources against Ascaris suum
Source: PLoS One. 2014 May 8;9(5):e97053. doi: 10.1371/journal.pone.0097053 (PMC4014605; doi:10.1371/journal.pone.0097053)

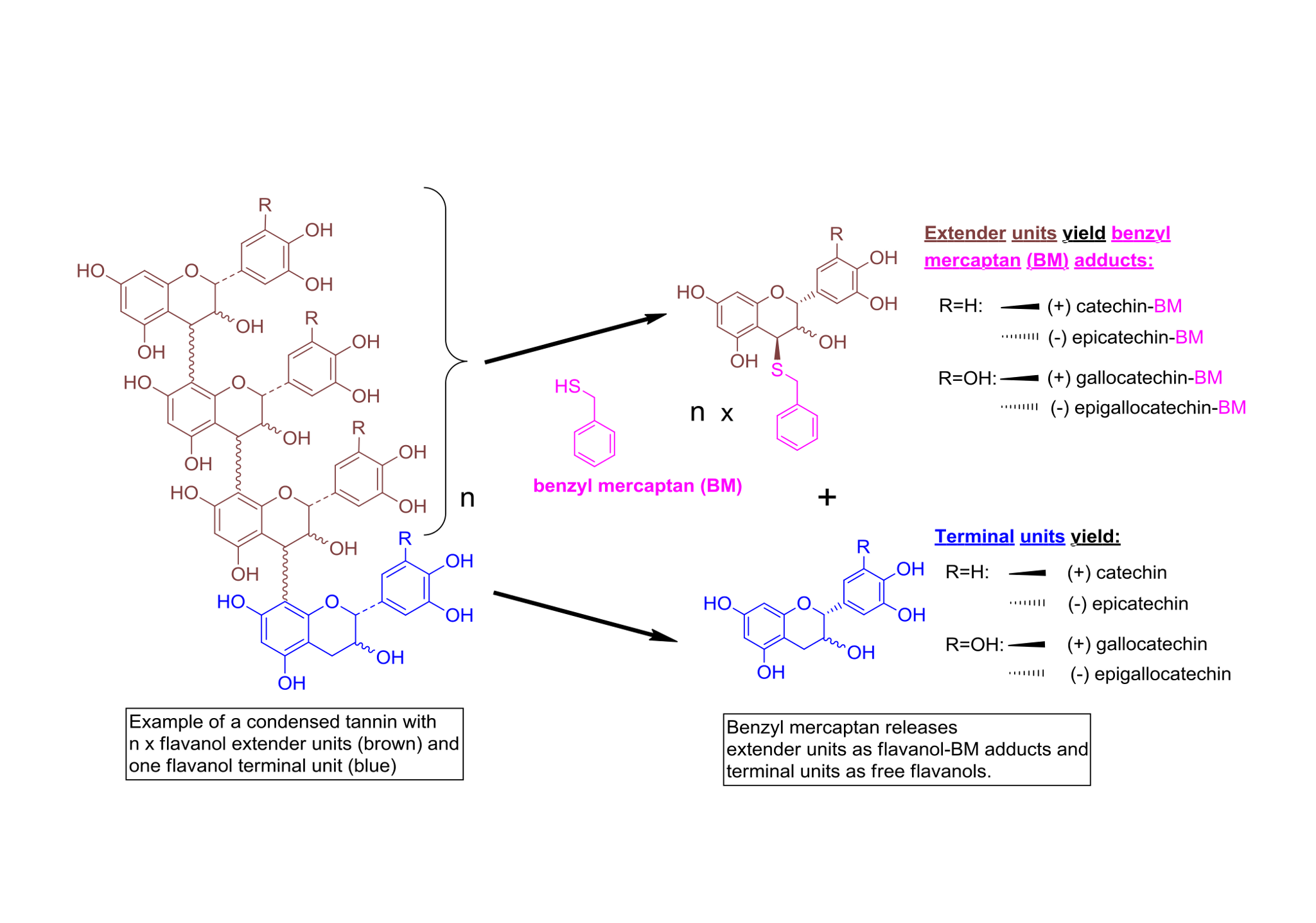

Supplement: Figure S1 — Explanation of thiolytic degradation scheme for determining chemical composition of condensed tannin molecules. (TIF) [file pone.0097053.s001.tif]

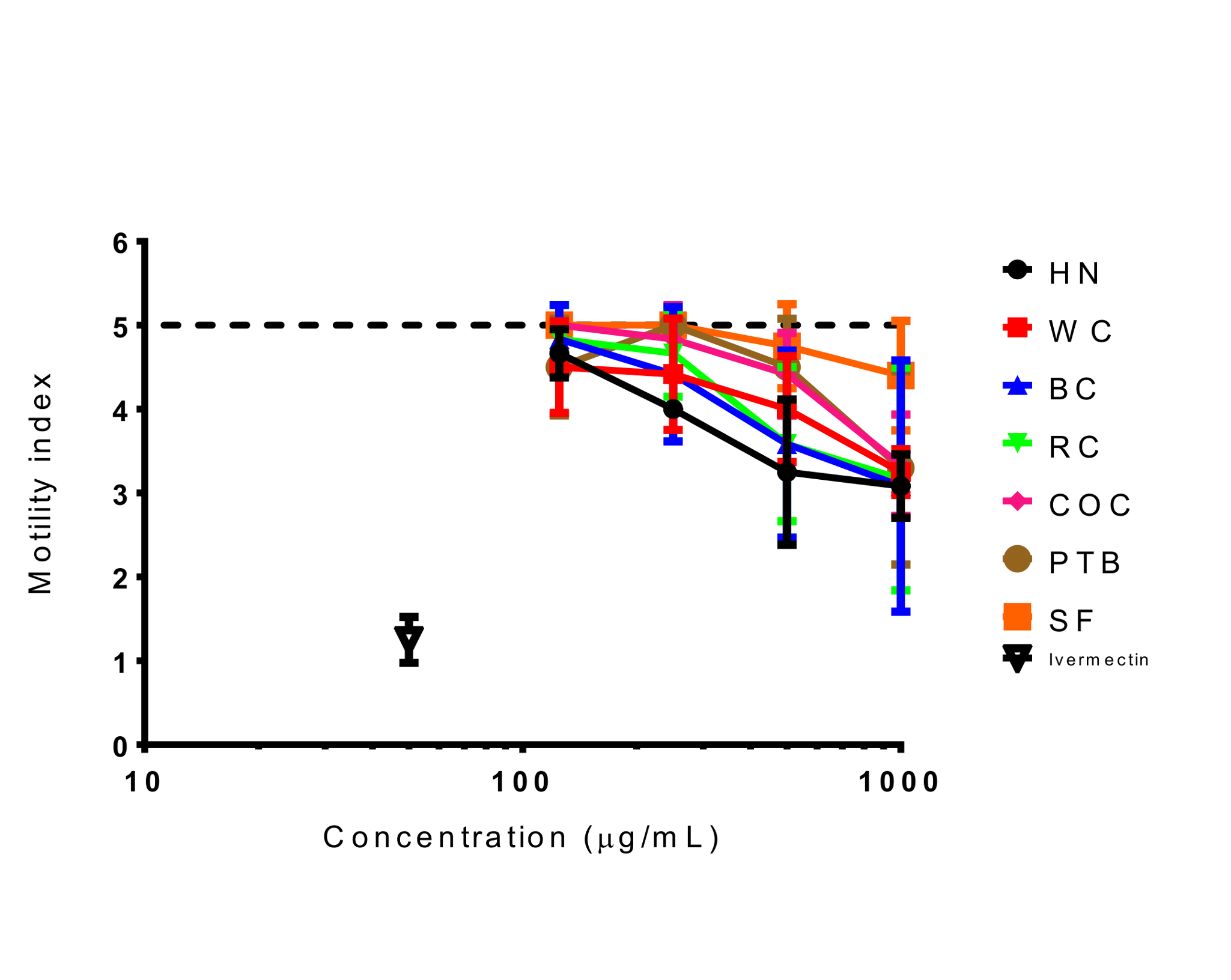

Supplement: Figure S2 — Motility of Ascaris suum L3 after exposure to extracts from tannin-containing plants. Motility of Ascaris suum L3 after 16 hours exposure to tannin-containing acetone/water extracts from hazelnut skins (HN), cocoa (COC), pine tree bark (PTB), sainfoin (SF), blackcurrant leaves (BC), redcurrant (RC) leaves and white clover flowers (WC). Motility is scored on a 0–5 scale where 5 is completely motile and 0 is completely still (see materials and methods). Dashed black line indicates the motility of larvae exposed to only culture medium (negative control). IVM = ivermectin at 50 µg/mL (positive control). Data points represent the mean of two independent experiments, each performed in triplicate. Error bars represent SEM of the individual replicates. (TIF) [file pone.0097053.s002.tif]

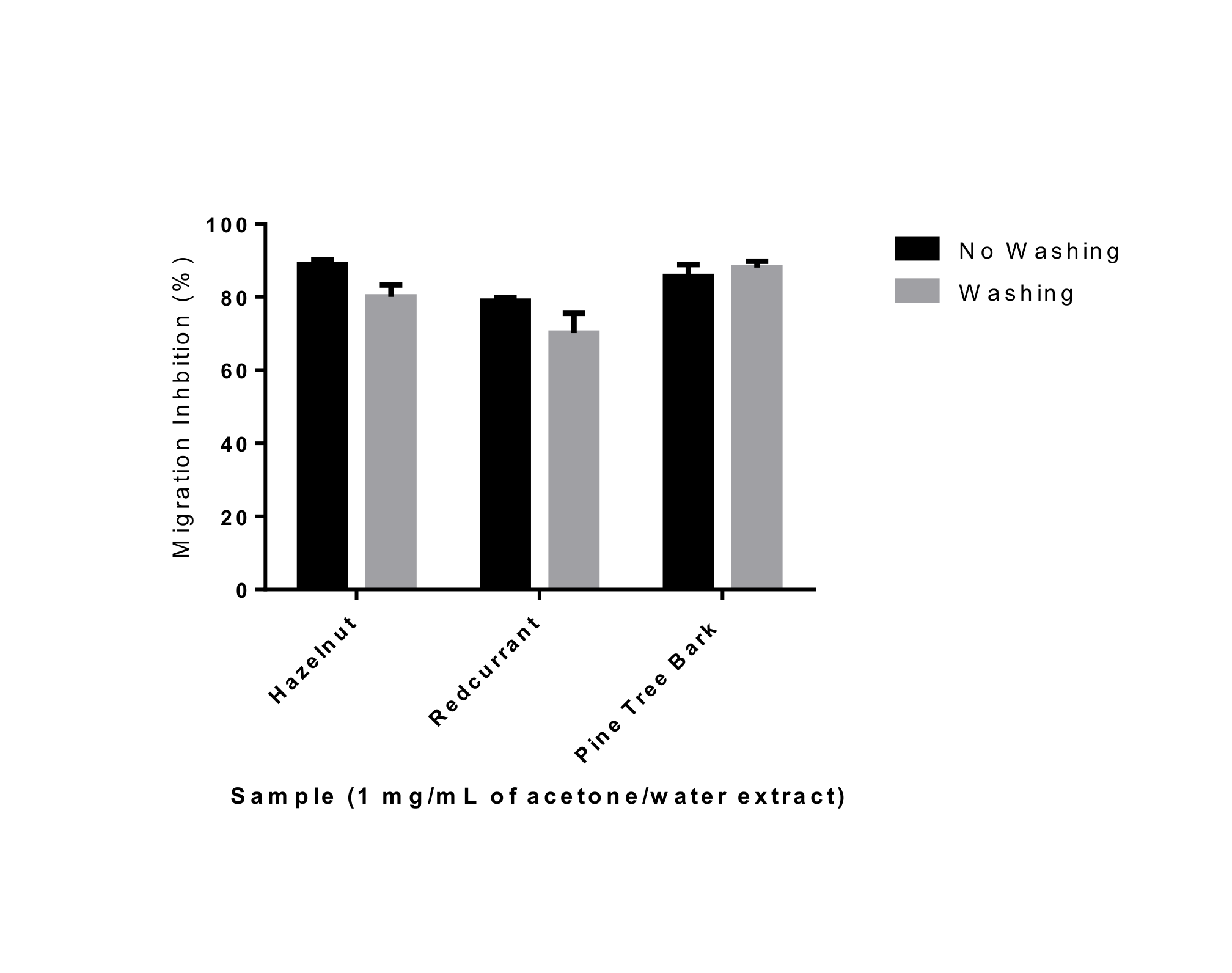

Supplement: Figure S3 — Effect of washing on inhibition of migratory activity (MIA). Ascaris suum L3 were incubated for 16 hours in tannin-containing acetone/water extracts from hazelnut skins, redcurrant leaves or pine bark. Larvae were then washed to remove tannins and resuspend in fresh media before addition of agar (‘washing’), or agar was added directly to the larvae in the tannin-containing media (‘no washing’). (TIF) [file pone.0097053.s003.tif]

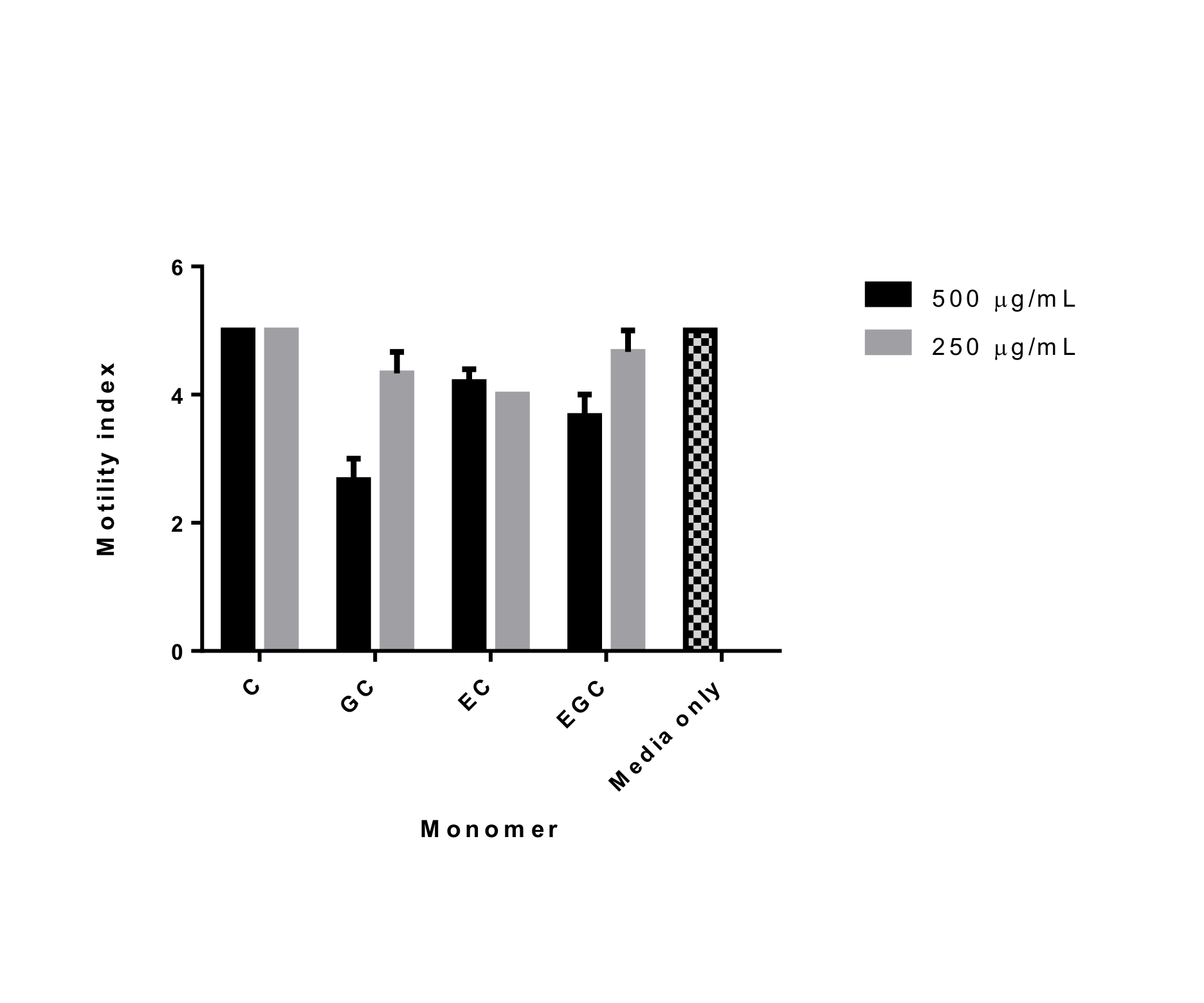

Supplement: Figure S4 — Motility of Ascaris suum L3 after exposure to flavanol monomers. Larvae were incubated for 16 hours with either catechin (C), gallocatechin (GC), epicatechin (EC) or epigallocatechin (EGC). Motility is scored on a 0–5 scale where 5 is completely motile and 0 is completely still (see materials and methods). Data points represent the mean of two independent experiments, each performed in triplicate. Error bars represent SEM of the individual replicates. (TIF) [file pone.0097053.s004.tif]

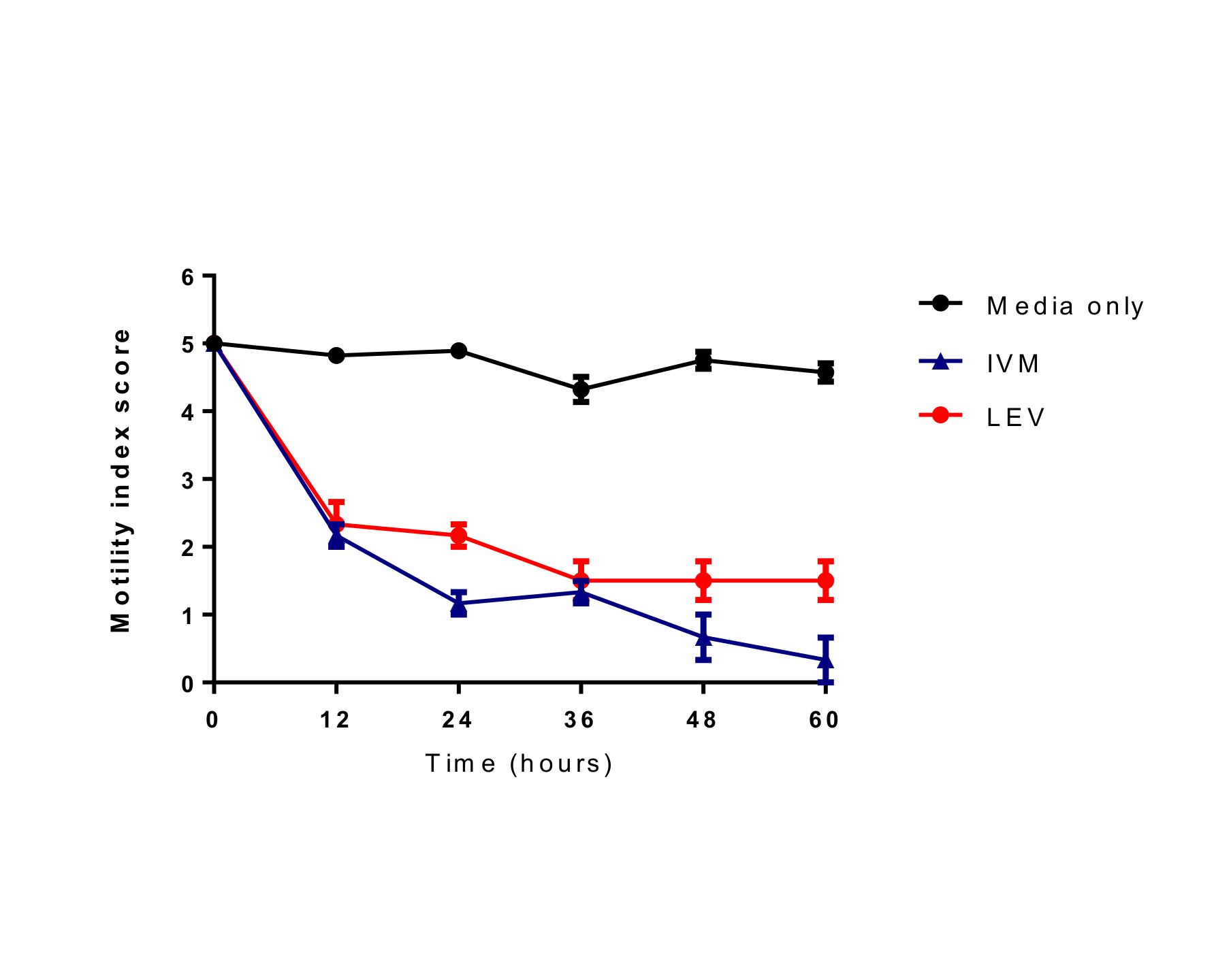

Supplement: Figure S5 — Motility of Ascaris suum L4 exposed to synthetic anthelmintic drugs. Motility of Ascaris suum L4 exposed to either 100 µg/mL ivermectin (IVM) or levamisole (LVM), or culture media only. Data points represent the mean of triplicate wells, with the error bars representing the inter-well SEM. (TIF) [file pone.0097053.s005.tif]
