# Supplementary material for: Direct Anthelmintic Effects of Condensed Tannins from Diverse Plant Sources against Ascaris suum
Source: PLoS One. 2014 May 8;9(5):e97053. doi: 10.1371/journal.pone.0097053 (PMC4014605; doi:10.1371/journal.pone.0097053)
Supplement: Table S1 — Proportions of monomeric flavanol subunits in extracts and fractions. Proportions of gallocatechin (GC), epigallocatechin (EGC), catechin (C) and epicatechin (EC) (DOCX) [file pone.0097053.s007.docx]

| **Sample** | **Extract or Fraction** | **% GC** | **%EGC** | **%C** | **%EC** |
| --- | --- | --- | --- | --- | --- |
| **Sainfoin** | A0 | 15.0 | 59.7 | 4.8 | 20.5 |
|  | F1 | 25.0 | 47.0 | 8.3 | 19.8 |
|  | F2 | 14.5 | 50.3 | 6.4 | 28.8 |
| **Cocoa beans** | A0 | 0.0 | 0.0 | 4.0 | 96.0 |
|  | F1 | 0.0 | 0.0 | 8.7 | 91.3 |
|  | F2 | 0.0 | 0.0 | 3.7 | 96.3 |
| **Pine bark** | A0 | 6.9 | 28.9 | 13.4 | 50.7 |
|  | F1 | 11.5 | 3.5 | 40.3 | 44.6 |
|  | F2 | 4.7 | 6.6 | 17.2 | 71.6 |
| **Hazelnut skin** | A0 | 10.7 | 9.8 | 39.5 | 39.9 |
|  | F1 | 10.9 | 7.4 | 48.1 | 33.7 |
|  | F2 | 11.7 | 9.2 | 40.5 | 38.6 |
| **Blackcurrant leaves** | A0 | 86.7 | 7.5 | 4.3 | 1.6 |
|  | F1 | 82.3 | 11.3 | 4.8 | 1.5 |
|  | F2 | 88.6 | 5.9 | 4.5 | 1.1 |
| **Redcurrant leaves** | A0 | 20.2 | 73.7 | 2.5 | 3.5 |
|  | F1 | 45.1 | 40.7 | 10.5 | 3.7 |
|  | F2 | 30.1 | 60.2 | 5.4 | 4.2 |
| **White clover flowers** | A0 | 33.9 | 65.3 | 0.4 | 0.4 |
|  | F2 | 40.4 | 58.3 | 0.7 | 0.6 |
